# Fine-Tuning the Biological Profile of Multitarget Mitochondriotropic Antioxidants for Neurodegenerative Diseases

**DOI:** 10.3390/antiox10020329

**Published:** 2021-02-23

**Authors:** Daniel Chavarria, Ophelie Da Silva, Sofia Benfeito, Sandra Barreiro, Jorge Garrido, Fernando Cagide, Pedro Soares, Fernando Remião, Xavier Brazzolotto, Florian Nachon, Paulo J. Oliveira, José Dias, Fernanda Borges

**Affiliations:** 1 CIQUP/Department of Chemistry and Biochemistry, Faculty of Sciences, University of Porto, 4169-007 Porto, Portugal; daniel.chavarria@fc.up.pt (D.C.); ester.benfeito@fc.up.pt (S.B.); jjg@isep.ipp.pt (J.G.); fernando.fagin@fc.up.pt (F.C.); pedro.soares@fc.up.pt (P.S.); 2 Département de Toxicologie et Risques Chimiques, Institut de Recherche Biomédicale des Armées, 91223 Brétigny-sur-Orge, France; ophelie1.da-silva@def.gouv.fr (O.D.S.); xavier.brazzolotto@def.gouv.fr (X.B.); florian.nachon@def.gouv.fr (F.N.); jose.dias@def.gouv.fr (J.D.); 3 UCIBIO-REQUIMTE, Laboratory of Toxicology, Department of Biological Sciences, Faculty of Pharmacy, University of Porto, 4050-313 Porto, Portugal; sandrafcbarreiro@gmail.com (S.B.); remiao@ff.up.pt (F.R.); 4 CIQUP/Department of Chemical Engineering, School of Engineering (ISEP), Polytechnic of Porto, 4200-072 Porto, Portugal; 5 CNC—Center for Neuroscience and Cell Biology, University of Coimbra, UC Biotech, Biocant Park, 3060-197 Cantanhede, Portugal; pauloliv@cnc.uc.pt

**Keywords:** neurodegenerative diseases, piperine, triphenylphosphonium, cholinesterases, monoamine oxidase, mitochondria-targeted antioxidants

## Abstract

Neurotransmitter depletion and mitochondrial dysfunction are among the multiple pathological events that lead to neurodegeneration. Following our previous studies related with the development of multitarget mitochondriotropic antioxidants, this study aims to evaluate whether the π-system extension on the chemical scaffolds of AntiOXCIN2 and AntiOXCIN3 affects their bioactivity and safety profiles. After the synthesis of four triphenylphosphonium (TPP^+^) conjugates (compounds **2**–**5**), we evaluated their antioxidant properties and their effect on neurotransmitter-metabolizing enzymes. All compounds were potent equine butyrylcholinesterase (*eq*BChE) and moderate electric eel acetylcholinesterase (*ee*AChE) inhibitors, with catechols 4 and 5 presenting lower IC_50_ values than AntiOXCIN2 and AntiOXCIN3, respectively. However, differences in the inhibition potency and selectivity of compounds **2**–**5** towards non-human and human cholinesterases (ChEs) were observed. Co-crystallization studies with compounds **2**–**5** in complex with human ChEs (*h*ChEs) showed that these compounds exhibit different binging modes to *h*AChE and *h*BChE. Unlike AntiOXCINs, compounds **2**–**5** displayed moderate human monoamine oxidase (*h*MAO) inhibitory activity. Moreover, compounds **4** and **5** presented higher ORAC-FL indexes and lower oxidation potential values than the corresponding AntiOXCINs. Catechols 4 and 5 exhibited broader safety windows in differentiated neuroblastoma cells than benzodioxole derivatives 2 and 3. Compound 4 is highlighted as a safe mitochondria-targeted antioxidant with dual ChE/MAO inhibitory activity. Overall, this work is a contribution for the development of dual therapeutic agents addressing both mitochondrial oxidative stress and neurotransmitter depletion.

## 1. Introduction

Neurodegeneration is a complex process that results from multiple mechanisms acting concurrently [1]. The main neuropathological hallmarks of Alzheimer’s disease (AD) and Parkinson’s Disease (PD) are the neuronal loss with consequent decrease of neurotransmitter levels, and the formation of protein aggregates [2,3]. Based on these observations, enzymes involved in neurotransmitters breakdown (e.g., cholinesterases (ChEs); monoamine oxidases (MAOs)) are among the main biological targets for the development of new therapeutics [1].

Mitochondria are also central players involved in the pathogenesis of AD and PD [4], since they are both one of the primary sources and one of the critical targets of reactive species (RS) [5]. Mitochondria play essential roles in the ATP synthesis, homeostasis of intracellular second messengers (calcium; RS), and apoptosis [6,7]. The high energy demand required for neuronal survival and excitability in the central nervous system (CNS) is mainly dependent on mitochondrial ATP generation [8]. Improper function of mitochondria may increase the neurons’ susceptibility to oxidative stress [9] and compromise neuronal survival [6]. Indeed, mitochondrial dysfunction is associated with increased RS production, intracellular calcium dyshomeostasis, and decreased ATP generation [4,10]. 

Considering the pivotal role of mitochondria in fundamental cellular processes [11], molecules that act on or accumulate in mitochondria may have great therapeutic potential [12]. To be able to act on mitochondrial targets, compounds usually need to be specifically directed towards these organelles [13]. One of the most commonly used strategies to deliver bioactive molecules to mitochondria is their conjugation with lipophilic cations such as triphenylphosphonium (TPP^+^) [13,14]. The selective accumulation of these lipophilic cations into the mitochondrial matrix occurs against the concentration gradient [15] and is driven by the plasma and mitochondrial membrane potentials [16]. 

Our research group recently developed mitochondria-targeted antioxidants, in which lipophilic TPP^+^ cations were conjugated with hydroxycinnamic (AntiOXCINs) and hydroxybenzoic (AntiOXBENs) acids [17,18,19,20,21,22]. We showed that these compounds accumulated within the mitochondrial matrix of rat liver mitochondria [18,19] and exhibited remarkable antioxidant properties [17,18,19,20,21]. Recently, we showed that they also displayed moderate to potent ChE inhibitory activities [17,22]. To understand the bioactivity and safety profiles of AntiOXCINs, we evaluated the effect of the modification of some of their substructures through structure-activity-toxicity studies. Accordingly, we evaluated the influence of the type of spacer between the phenolic ring and the carboxamide group (none, vinyl, methylene, ethylene), the length of the alkyl linker between the carboxamide group and the TPP^+^ moiety (six- or ten-carbon), and the substitution pattern of the phenolic ring (catechol or pyrogallol) [21,22]. 

**Figure 1:**
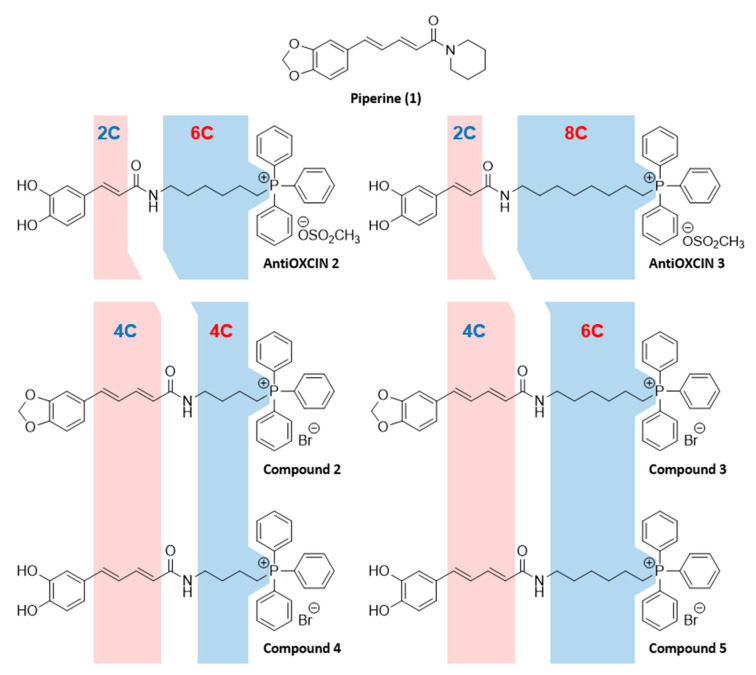
Rational design of mitochondria-targeted compounds **2**–**5** based on AntiOXCIN2, AntiOXCIN3, and piperine (compound **1**).

Within this framework, we aim to evaluate whether the elongation of the α,β-unsaturated chain of AntiOXCINs while maintaining the compounds’ overall lipophilicity affects their bioactivity and safety profiles. Interestingly, this type of substructure (5-phenyl-2,4-pentadienyl moiety) can be found in the chemical structure of piperine (1-[5-(1,3-benzodioxol-5-yl)-1-oxo-2,4-pentadienyl]piperidine), compound **1**, Figure 1), the main alkaloid found in numerous piper species [23]. Recent pharmacological studies in AD and PD animal models showed that piperine improves cognitive function [24,25], attenuates rotenone-induced motor function and mitigates neuronal loss in substantia nigra [26] and in the hippocampus [25]. Piperine also protected neurons against rotenone-induced mitochondrial damage [26] and oxidative stress [25,27], and presented MAO inhibition properties [28,29]. 

Taken together these plausible assumptions, herein we report the synthesis of four new TPP^+^ cations (compounds **2**–**5**, Figure 1) inspired on the chemical structures of AntiOXCIN2, AntiOXCIN3 and piperine. In general, the rational design consisted of introducing an additional ethylenic group, with a concomitant shortening of the alkyl linker between the carboxamide group and the TPP^+^ moiety (Figure 1). This strategy was used to obtain new catechol antioxidants (compounds **4** and **5**) with lipophilicity similar to AntiOXCIN2 and AntiOXCIN3. Due to the relevant biological activities of piperine, benzodioxole derivatives 2 and 3 were also included in this study. We then evaluated the compounds’ inhibition profile and binding mode towards cholinesterases (acetylcholinesterase (AChE, EC 3.1.1.7) and butyrylcholinesterase (BChE, EC 3.1.1.8)) and MAOs (MAO-A and MAO-B, EC 1.4.3.4), as well as their antioxidant and redox properties. Finally, we studied their cytotoxicity in differentiated neuroblastoma (SH-SY5Y) cells and predicted their ability to cross the blood-brain barrier.

## 2. Materials and Methods

### 2.1. Chemistry

The reagents, general methods and apparatus are described in supplementary information.

#### 2.1.1. Synthesis of AntiOXCIN2 and AntiOXCIN3

Synthesis and structural analysis were previously reported by Teixeira et al. [19].

#### 2.1.2. Synthesis of Mitochondria-Targeted Agents Inspired on Piperine

##### General Procedure for the Obtention of Phtalimidylalkylltriphenylphosphonium Salts

A mixture containing the appropriate *N*-(bromoalkyl)phthalimide (1 mmol) and triphenylphosphine (1.2 mmol) was mixed under argon atmosphere and heated at 130 °C, protected from the light. The solid obtained was recrystallized from dichloromethane/diethyl ether. The procedure was adapted from Cheng et al. 2016 [30] with some modifications. 

(4-(1,3-Dioxoisoindolin-2-yl)butyl)triphenylphosphonium bromide (8). Compound **8** was obtained in the following conditions: compound **6** (1.00 g, 3.56 mmol), triphenylphosphine (1.06 g, 4.03 mmol). The mixture was heated at 130 °C for 10 h. η = 97 %. ^1^H NMR (CDCl_3_-*d_1_*): δ (ppm) = 1.62 (*m*, 2H, N(CH_2_)_2_CH_2_CH_2_), 2.15 (*m*, 2H, NCH_2_CH_2_(CH_2_)_2_), 3.77 (*t*, *J* = 6.3 Hz, 2H, N(CH_2_)_3_CH_2_), 4.01 (*m*, 2H, NCH_2_(CH_2_)_3_), 7.71 (*m*, 13 H, PPh_3_, H5, H6, H7, H8), 7.85 (*m,* 6H, PPh_3_). ^13^C NMR (CDCl_3_-*d_1_*): δ (ppm) = 19.5 (*d*, *J* = 3.9 Hz, NCH_2_CH_2_(CH_2_)_2_), 21.7 (*d*, *J* = 50.4 Hz, N(CH_2_)_3_CH_2_), 28.6 (*d*, *J* = 16.8 Hz, N(CH_2_)_2_CH_2_CH_2_), 36.4 (NCH_2_(CH_2_)_3_), 118.3 (*d*, *J* = 86.0 Hz, C1’), 123.2 (C5, C8), 130.4 (*d*, *J* = 12.5 Hz, C3’, C5’), 131.9 (C4, C9), 133.8 (*d*, *J* = 9.9 Hz, C2’, C6’), 134.1 (C6, C7), 135.0 (*d*, *J* = 2.9 Hz, C4’), 168.4 (C1, C3). ESI/MS m/z (%): 464 (M^+^-Br, 100).

(6-(1,3-Dioxoisoindolin-2-yl)hexyl)triphenylphosphonium bromide (9). Compound 9 was obtained in the following conditions: compound 7 (0.50 g, 1.61 mmol), triphenylphosphine (0.63 g, 2.42 mmol). The mixture was heated at 130 °C for 1.5 h. η = 87 %. ^1^H NMR (CDCl_3_-*d_1_*): δ (ppm) = 1.32 (*m*, 2H, NCH_2_CH_2_(CH_2_)_4_), 1.61 (*m*, 4H, N(CH_2_)_2_CH_2_(CH_2_)_3_, N(CH_2_)_3_CH_2_(CH_2_)_2_), 1.72 (*m*, 2H, N(CH_2_)_4_CH_2_CH_2_)), 3.62 (*t*, *J* = 7.0 Hz, 2H, NCH_2_(CH_2_)_5_), 3.87 (*m,* 2H, N(CH_2_)_5_CH_2_), 7.77 (*m*, 19H, PPh_3_, H5, H6, H7, H8). ^13^C NMR (CDCl_3_-*d_1_*): δ (ppm) = 22.1 (*d*, *J* = 4.9 Hz, N(CH_2_)_3_CH_2_(CH_2_)_2_), 22.8 (*d*, *J* = 49.1 Hz, N(CH_2_)_5_CH_2_), 26.5 (N(CH_2_)_2_CH_2_(CH_2_)_3_), 28.2 (NCH_2_CH_2_(CH_2_)_4_), 29.8 (*d*, *J* = 15.7 Hz, N(CH_2_)_4_CH_2_CH_2_), 37.8 (NHCH_2_(CH_2_)_5_), 118.5 (*d*, *J* = 85.8 Hz, C1’), 123.2 (C5, C8), 130.5 (*d*, *J* = 12.5 Hz, C3’, C5’), 132.2 (C4, C9), 133.8 (*d*, *J* = 10.1 Hz, C2’, C6’), 133.9 (C6, C7), 134.9 (*d*, *J* = 2.9 Hz, C4’), 168.4 (C1, C3). ESI/MS m/z (%): 492 (M^+^-Br, 100).

##### General Procedure for the Obtention of Aminoalkylltriphenylphosphonium Salts 

In a round bottom flask with compounds **8** or **9** (1 mmol) dissolved in ethanol (5 mL), *n*-butylamine (10–24 mmol) was added. The mixture was refluxed until the reaction was complete. Then, the solvent was partially concentrated, and water was added. The solid obtained was filtered off and the filtrate was extracted with dichloromethane. Then, the aqueous phase was concentrated. The material was used without further purification in the next step. The procedure was adapted from Cheng et al. [30] 2016 with some modifications.

(4-Aminobutyl)triphenylphosphonium bromide (10). Compound 10 was obtained in the following conditions: compound 8 (0.93 g, 1.71 mmol), ethanol (10 mL), *n*-butylamine (2.2 mL, 40.6 mmol). The mixture was refluxed for 10 h. η = 86 %. ^1^H NMR (MeOD-*d_4_*): δ (ppm) = 1.74 (*m*, 4H, NH_2_CH_2_CH_2_(CH_2_)_2_, NH_2_(CH_2_)_2_CH_2_CH_2_), 2.73 (*t*, *J* = 6.8 Hz, 2H, NH_2_CH_2_(CH_2_)_3_), 3.44 (*m,* 2H, NH_2_(CH_2_)_3_CH_2_), 7.77 (*m*, 15H, PPh_3_). ^13^C NMR (MeOD-*d_4_*): δ (ppm) = 19.7 (*d*, *J* = 4.2 Hz, NH_2_CH_2_CH_2_(CH_2_)_2_), 21.3 (*d*, *J* = 51.5 Hz, NH_2_(CH_2_)_3_CH_2_), 31.8 (*d*, *J* = 16.8 Hz, NH_2_(CH_2_)_2_CH_2_CH_2_), 39.8 (NH_2_CH_2_(CH_2_)_3_), 118.5 (*d*, *J* = 86.6 Hz, C1’), 130.2 (*d*, *J* = 12.6 Hz, C3’, C5’), 133.5 (*d*, *J* = 10.1 Hz, C2’, C6’), 134.9 (*d*, *J* = 3.1 Hz, C4’). ESI/MS m/z (%): 334 (M^+^-Br, 100).

(6-Aminohexyl)triphenylphosphonium bromide (11). Compound 11 was obtained in the following conditions: compound 9 (2.00 g, 3.49 mmol), ethanol (24 mL), butylamine (3.5 mL, 34.9 mmol). The mixture was refluxed for 1.5 h. η = 80 %. ^1^H NMR (MeOD-*d_4_*): δ (ppm) = 1.44 (*m*, 2H, NHCH_2_CH_2_(CH_2_)_4_), 1.68 (*m*, 6H, NH_2_(CH_2_)_2_CH_2_(CH_2_)_3_, NH_2_(CH_2_)_3_CH_2_(CH_2_)_2_, NH_2_(CH_2_)_4_CH_2_CH_2_)), 2.92 (*t*, *J* = 6.8 Hz, 2H, NH_2_CH_2_(CH_2_)_5_), 3.42 (*m,* 2H, NH_2_(CH_2_)_5_CH_2_), 7.84 (*m*, 15H, PPh_3_). ^13^C NMR (MeOD-*d_4_*): δ (ppm) = 21.3 (*d*, *J* = 51.4 Hz, NH_2_(CH_2_)_5_CH_2_), 22.0 (*d*, *J* = 4.3 Hz, NH_2_(CH_2_)_3_CH_2_(CH_2_)_2_), 25.3 (NH_2_(CH_2_)_2_CH_2_(CH_2_)_3_), 26.9 (NH_2_CH_2_CH_2_(CH_2_)_4_), 29.6 (*d*, *J* = 16.6 Hz, NH(CH_2_)_4_CH_2_CH_2_), 39.1 (NHCH_2_(CH_2_)_5_), 118.5 (*d*, *J* = 86.4 Hz, C1’), 130.2 (*d*, *J* = 12.6 Hz, C3’, C5’), 133.4 (*d*, *J* = 9.9 Hz, C2’, C6’), 134.9 (*d*, *J* = 3.0 Hz, C4’). ESI/MS m/z (%): 362 (M^+^-Br, 100).

##### Synthesis of (*2E,4E*)-5-(Benzo[d][1,3]dioxol-5-yl)Penta-2,4-Dienoic Acid (12)

Synthesis and structural analysis described in literature [29].

##### Piperic Acid Amidation

Piperic acid (compound 2, 1 mmol) was dissolved in dichloromethane (2.5 mL) and Et_3_N (2 mmol). To the stirred solution kept in an ice bath, ethyl chloroformate (2 mmol) was added dropwise. After stirring for 2 h at room temperature, the mixture was cooled again in an ice bath and the appropriate amine (compounds 10 or 11, 2 mmol) was slowly added. The reaction was stirred overnight at room temperature. Upon completion, dichloromethane (30 mL) was added. The mixture was extracted with HBr 1 M (3 × 10 mL) and washed with water. The combined organic phases were dried over anhydrous Na_2_SO_4_, filtered and concentrated. The crude product was purified by flash column chromatography (silica, dichloromethane/methanol (9:1)) and recrystallized from methanol/water. The procedure was adapted from Teixeira et al. [20] with some modifications. 

(4-((*2E,4E*)-5-(Benzo[*d*][1,3]dioxol-5-yl)penta-2,4-dienamido)butyl)triphenylphosphonium bromide (2). Compound 2 was obtained in the following conditions: compound 12 (0.42 g, 1.93 mmol), dichloromethane (8 mL), Et_3_N (700 µL, 3.65 mmol), ethyl chloroformate (362 µL, 4.18 mmol), amine 10 (1.09 g, 2.46 mmol). η = 44 %. ^1^H NMR (CDCl_3_-*d_1_*): δ (ppm) = 1.76 (*m*, 2H, NHCH_2_CH_2_(CH_2_)_2_), 1.98 (*m*, 2H, NH(CH_2_)_2_CH_2_CH_2_), 3.47 (*dd*, *J* = 11.7 Hz, 5.8 Hz, 2H, NHCH_2_(CH_2_)_3_), 3.65 (*m,* 2H, NH(CH_2_)_3_CH_2_), 5.98 (*s*, 2H, OCH_2_O), 6.43 (*d, J* = 15.0 Hz, 1H, Hα), 6.77 (*m*, 3H, H5, Hγ, Hδ), 6.90 (*dd*, *J* = 1.6 Hz, 8.2 Hz, 1H, H6), 6.99 (*d, J* = 1.6 Hz, 1H, H2), 7.31 (*dd, J =* 10.4 Hz, 15.0 Hz, 1H, Hβ), 7.74 (*m*, 15 H, PPh_3_), 8.55 (*t*, *J* = 5.6 Hz, 1H, CONH). ^13^C NMR (CDCl_3_-*d_1_*): δ (ppm) = 19.5 (*d*, *J* = 4.0 Hz, NHCH_2_CH_2_(CH_2_)_2_), 22.4 (*d*, *J* = 50.4 Hz, NH(CH_2_)_3_CH_2_), 28.7 (*d*, *J* = 16.6 Hz, NH(CH_2_)_2_CH_2_CH_2_), 37.4 (NHCH_2_(CH_2_)_3_), 101.2 (OCH_2_O), 105.9 (C2), 108.4 (C5), 118.2 (*d*, *J* = 86.0 Hz, C1’), 122.2 (Cα), 125.0 (C6), 125.7 (Cγ), 130.5 (*d*, *J* = 12.5 Hz, C3’, C5’), 131.3 (C1), 133.7 (*d*, *J* = 10.0 Hz, C2’, C6’), 135.1 (*d*, *J* = 2.9 Hz, C4’), 137.5 (Cδ), 139.5 (Cβ), 147.9 (C3), 148.2 (C4), 167.3 (CONH). ESI/MS m/z (%): 534 (M^+^-Br, 100).

(6-((*2E,4E*)-5-(Benzo[*d*][1,3]dioxol-5-yl)penta-2,4-dienamido)hexyl)triphenylphosphonium bromide (3). Compound 3 was obtained in the following conditions: compound 12 (0.76 g, 3.48 mmol), dichloromethane (20 mL), Et_3_N (1 mL, 7.12 mmol), ethyl chloroformate (665 µL, 6.96 mmol), amine 11 (2.00 g, 4.52 mmol). η = 46 %. ^1^H NMR (CDCl_3_-*d_1_*): δ (ppm) = 1.60 (*m*, 8H, NHCH_2_CH_2_(CH_2_)_4_, NH(CH_2_)_2_CH_2_(CH_2_)_3_, NH(CH_2_)_3_CH_2_(CH_2_)_2_, NH(CH_2_)_4_CH_2_CH_2_), 3.37 (*m*, 2H, NHCH_2_(CH_2_)_5_), 3.68 (*m*, 2H, NH(CH_2_)_5_CH_2_), 5.97 (*s*, 2H, OCH_2_O), 6.61 (*d, J* = 15.0 Hz, 1H, Hα), 6.67 (*d, J* = 15.6 Hz, 1H, Hδ), 6.76 (*m*, 2H, H5, Hγ), 6.86 (*dd*, *J* = 1.6 Hz, 8.2 Hz, 1H, H6), 6.95 (*d, J* = 1.6 Hz, 1H, H2), 7.34 (*dd, J =* 11.6 Hz, 15.0 Hz, 1H, Hβ), 7.71 (*m*, 6H, PPh_3_), 7.82 (*m*, 9H, PPh_3_), 8.24 (*t*, *J* = 4.9 Hz, 1H, CONH). ^13^C NMR (CDCl_3_-*d_1_*): δ (ppm) = 22.1 (*d*, *J* = 4.4 Hz, NH(CH_2_)_3_CH_2_(CH_2_)_2_), 22.3 (*d*, *J* = 50.2 Hz, NH(CH_2_)_5_CH_2_), 25.5 (NH(CH_2_)_2_CH_2_(CH_2_)_3_), 28.4 (NHCH_2_CH_2_(CH_2_)_4_), 29.2 (*d*, *J* = 16.2 Hz, NH(CH_2_)_4_CH_2_CH_2_), 38.7 (NHCH_2_(CH_2_)_5_), 101.2 (OCH_2_O), 105.8 (C2), 108.4 (C5), 118.3 (*d*, *J* = 85.8 Hz, C1’), 122.2 (Cα), 125.2 (C6), 126.0 (Cγ), 130.5 (*d*, *J* = 12.5 Hz, C3’, C5’), 131.4 (C1), 133.6 (*d*, *J* = 10.0 Hz, C2’, C6’), 135.1 (*d*, *J* = 2.9 Hz, C4’), 137.3 (Cδ), 139.6 (Cβ), 147.8 (C3), 148.1 (C4), 167.1 (CONH). ESI/MS m/z (%): 562 (M^+^-Br, 100).

##### Demethylenation Reaction

In a round bottom flask, compounds 2 or 3 (1 mmol) were maintained under argon atmosphere. Then, the compounds were dissolved in anhydrous dichloromethane and BBr_3_.S(CH_3_)_2_ (4 mmol) was added. The mixture was protected from the light and refluxed for 6 h. Upon completion, water was added to destroy BBr_3_.S(CH_3_)_2_. The solid formed was isolated by filtration and purified by flash column chromatography (silica, dichloromethane/methanol (95:5)). The procedure was adapted from Williard et al. [31] with some modifications.

(4-((*2E,4E*)-5-(3,4-Dihydroxyphenyl)penta-2,4-dienamido)butyl)triphenylphosphonium bromide (4). Compound 4 was obtained in the following conditions: compound 2 (1.28 g, 2.10 mmol), dichloromethane (35 mL), BBr_3_.S(CH_3_)_2_ (2.85 g, 9.1 mmol). η = 29 %. ^1^H NMR (DMSO-*d_6_*): 1.55 (*m*, 2H, NH(CH_2_)_2_CH_2_CH_2_), 1.64 (*m*, 2H, NHCH_2_CH_2_(CH_2_)_2_), 3.77 (*dd*, *J* = 3.7 Hz, 7.8 Hz, 2H, NH(CH_2_)_3_CH_2_), 4.01 (*m*, 2H, NHCH_2_(CH_2_)_3_), 5.98 (*d, J* = 15.0 Hz, 1H, Hα), 6.74 (*m,* 3H, Hδ, H5, Hγ), 6.85 (*dd*, *J* = 2.0 Hz, 8.2 Hz, 1H, H6), 6.95 (*d, J* = 1.9 Hz, 1H, H2), 7.12 (*dd, J =* 10.2 Hz, 15.0 Hz, 1H, Hβ), 7.78 (*m*, 12H, PPh_3_), 7.89 (*m*, 3H, PPh_3_), 8.00 (*t*, *J* = 5.4 Hz, 1H, CONH), 9.11 (*s*, 2H, 2×OH). ^13^C NMR (DMSO-*d_6_*): δ (ppm) = 19.1 (*d*, *J* = 3.9 Hz, NHCH_2_CH_2_(CH_2_)_2_), 19.8 (*d*, *J* = 50.1 Hz, NH(CH_2_)_3_CH_2_), 29.8 (*d*, *J* = 16.9 Hz, NH(CH_2_)_2_CH_2_CH_2_), 37.1 (NCH_2_(CH_2_)_3_), 113.7 (C2), 115.6 (C5), 118.4 (*d*, *J* = 85.7 Hz, C1’), 119.3 (Cα), 123.2 (C6), 123.4 (Cγ), 127.8 (C1), 130.2 (*d*, *J* = 12.4 Hz, C3’, C5’), 133.5 (*d*, *J* = 10.1 Hz, C2’, C6’), 134.8 (*d*, *J* = 2.5 Hz, C4’), 138.7 (Cδ), 139.6 (Cβ), 145.4 (C3), 146.5 (C4), 165.4 (CONH). ESI/MS m/z (%): 522 (M^+^-Br, 100).

(6-((*2E,4E*)-5-(3,4-Dihydroxyphenyl)penta-2,4-dienamido)hexyl)triphenylphosphonium bromide (5). Compound 5 was obtained in the following conditions: compound 3 (1.11 g, 1.72 mmol), dichloromethane (30 mL), BBr_3_.S(CH_3_)_2_ (2.35 g, 7.52 mmol). η = 44 %. ^1^H NMR (DMSO-*d_6_*): δ (ppm) = 1.28 (*m*, 2H, NH(CH_2_)_2_CH_2_(CH_2_)_3_), 1.38 (*m*, 2H, NH(CH_2_)_3_CH_2_(CH_2_)_2_), 1.49 (*m*, 4H, NHCH_2_CH_2_(CH_2_)_4_, NH(CH_2_)_4_CH_2_CH_2_), 3.11 (*m*, 2H, NHCH_2_(CH_2_)_5_), 3.58 (*m*, 2H, NH(CH_2_)_5_CH_2_), 6.03 (*d, J* = 15.0 Hz, 1H, Hα), 6.71 (*m,* 3H, Hδ, H5, Hγ), 6.83 (*dd*, *J* = 2.0 Hz, 8.3 Hz, 1H, H6), 6.94 (*d, J* = 2.0 Hz, 1H, H2), 7.11 (*dd, J =* 9.8 Hz, 15.1 Hz, 1H, Hβ), 7.79 (*m*, 12H, PPh_3_), 7.90 (*m*, 3H, PPh_3_), 7.94 (*t*, *J* = 5.6 Hz, 1H, CONH), 8.98 (*s*, 1H, OH), 9.27 (*s*, 1H, OH). ^13^C NMR (DMSO-*d_6_*): δ (ppm) = 20.1 (*d*, *J* = 49.9 Hz, NH(CH_2_)_5_CH_2_), 21.6 (*d*, *J* = 4.3 Hz, NH(CH_2_)_3_CH_2_(CH_2_)_2_), 25.5 (NH(CH_2_)_2_CH_2_(CH_2_)_3_), 28.8 (NHCH_2_CH_2_(CH_2_)_4_), 29.4 (*d*, *J* = 16.7 Hz, NH(CH_2_)_4_CH_2_CH_2_), 38.3 (NHCH_2_(CH_2_)_5_), 113.6 (C2), 115.6 (C5), 118.5 (*d*, *J* = 85.6 Hz, C1’), 119.2 (Cα), 123.5 (C6), 123.5 (Cγ), 127.8 (C1), 130.1 (*d*, *J* = 12.4 Hz, C3’, C5’), 133.5 (*d*, *J* = 10.1 Hz, C2’, C6’), 134.8 (*d*, *J* = 2.8 Hz, C4’), 138.4 (Cδ), 139.3 (Cβ), 145.4 (C3), 146.4 (C4), 165.1 (CONH). ESI/MS m/z (%): 550 (M^+^-Br, 100).

### 2.2. Enzymatic Assays 

#### 2.2.1. Acetylcholinesterase and Butyrylcholinesterase

##### Evaluation of Eel Acetylcholinesterase and Equine Butyrylcholinesterase Inhibitory Activity

The *ee*AChE and *eq*BChE inhibitory activities of the compounds under study were determined following the Ellman’s method [32,33] (see supplementary information). 

##### Evaluation of Human Acetylcholinesterase and Human Butyrylcholinesterase Inhibitory Activities

Recombinant *h*AChE and *h*BChE were produced and purified as previously described [34,35]. Test compounds were dissolved in methanol 100 % to prepare stock solutions at 40 mM and subsequently diluted in water to reach the desired concentrations. Recombinant *h*AChE and *h*BChE activities were measured in presence of appropriate compound concentrations by spectrophotometry at 412 nm and 25 °C in a 1 mL cuvette containing Ellman’s buffer (0.5 mM DTNB, 0.1% BSA, 0.1 M phosphate, pH 7.4). Measurements were performed at least in duplicate for each tested concentration and final methanol concentrations were kept below 5%. The compound concentration producing 50 % inhibition was determined by nonlinear fitting with ProFit (Quantumsoft) using Equation (1).

(1) $$\%\;\mathrm{Activity}\;=\;\frac{{100\;\times \;\mathrm{IC}}_{50}}{{(\mathrm{IC}}_{50}+\left[\mathrm{Cp}\right])}\;$$

##### Crystallization, X-ray Data Collection and Processing

Recombinant *h*AChE and *h*BChE crystals were grown by hanging drop vapor diffusion at 20 °C. For *h*AChE, a crystallization buffer containing lithium sulfate 1.6 M, HEPES 100 mM pH 7 and magnesium sulfate 60 mM was used. For *h*BChE, the crystallization buffer was MES 0.1 M pH 6.5 and ammonium sulfate 2.15 M. Crystals of *h*AChE and *h*BChE were soaked overnight in presence of compounds 2–5 at 1 mM. Crystals were then washed with a cryoprotectant solution composed of the crystallization buffer with glycerol 20% and flash-cooled in liquid nitrogen. 

Diffraction data were collected at the Proxima 2 beam line, synchrotron SOLEIL (Gif-sur-Yvette), at 100 K on an EIGER X 9M area detector (λ = 0.9801 Å). Data were processed with XDS [36] and scaled with XSCALE. The structures were solved by molecular replacement with PHASER [37] using PDB 4EY4 as a starting model for *h*AChE and PDB 1P0I for *h*BChE. The models were built by iterative cycles of model building using Coot [38] and refinement using Phenix [39]. Geometry restrains of compound 3 were generated using Phenix.eLBOW and the AM1 semi-empirical quantum mechanical method. Table 1 shows the crystallographic data collection and refinement statistics of the *h*AChE (PDB: 6ZWE) and the *h*BChE (PDB: 6ZWI) in complex with compound 3.

#### 2.2.2. Evaluation of Human Monoamine Oxidase Inhibitory Activity

The inhibitory activity of lipophilic TPP^+^ cations on *h*MAO-A and *h*MAO-B was studied using an experimental protocol described elsewhere [29,40] (see supplementary information). 

### 2.3. Oxygen Radical Absorbance Capacity (ORAC-FL) Assay

The ORAC-FL assay was performed using an experimental protocol adapted from the literature [41,42] and described in Supplementary Information.

### 2.4. Electrochemical Measurements

DPV experiments were performed as described in literature [43] (see supplementary information).

### 2.5. In Vitro Toxicology

#### 2.5.1. Materials

All reagents used were of analytical grade or of the highest grade available. Neutral red (NR) solution, resazurin, Dulbecco’s Modified Eagle’s Medium (DMEM) with 4.5 g/L glucose, retinoic acid and 12-*O*-tetradecanoylphorbol-13-acetate (TPA) were obtained from Sigma Aldrich. Reagents used in cell culture such as heat-inactivated fetal bovine serum (FBS), antibiotic (10,000 U/mL penicillin, 10,000 μg/mL streptomycin), MEM Non-Essential Amino Acids solution (100×) (MEM NEAA), 0.25% trypsin/1 mM ethylenediamine tetraacetic acid (EDTA) and Hanks’ balanced salt solution (HBSS) were purchased from Gibco Laboratories (Lenexa, KS, USA). Dimethylsulfoxide (DMSO), absolute ethanol, and acetic acid were obtained from Merck (Darmstadt, Germany).

#### 2.5.2. Cell Lines and Culture Conditions

Human SH-SY5Y neuroblastoma cells and human epithelial colorectal adenocarcinoma (Caco-2) cells were obtained from the American Type Culture Collection (ATCC, Manassas, VA, USA). Cell culture and cell differentiation were performed as previously described by Fernandes et al. [44] and in supplementary information.

#### 2.5.3. Cytotoxicity

Stock solutions of the test compounds (100 mM) were freshly prepared in DMSO. Final concentrations of the test compounds were obtained by diluting them into cell culture medium immediately before use, giving a final maximum concentration of 0.1% DMSO.

For cytotoxicity studies, differentiated SH-SY5Y cells were incubated with increasing concentrations of the test compounds (0.20–100 µM) for 24 h. Controls were treated with culture media containing 0.1% DMSO. Cell viability was estimated using two different methods: resazurin reduction assay and NR uptake assay. 

#### 2.5.4. Statistical Analysis 

The data obtained are expressed as mean ± standard error mean (SEM) of at least three independent experiments (*n* = 3). All statistical analyses were performed using GraphPad PRISM version 6 for Windows. The normality of the data distribution was evaluated using three normality tests: KS normality test, D’Agostino and Pearson omnibus normality test, and Shapiro−Wilk normality test. Statistical comparisons between groups were estimated using the parametric method two-way analysis of variance (ANOVA) followed by Dunnett’s multiple comparison test. In all cases, *p* values lower than 0.05 were considered significant.

### 2.6. Evaluation of the Chromatographic Hydrophobicity Index

Chromatographic hydrophobicity indexes (CHIs) at pH 2.3 were determined using an experimental protocol described elsewhere [45,46] and included in the supplementary information.

### 2.7. Estimation of Drug-Like Properties

The calculation of molecular weight (MW), topological polar surface area (TPSA), number of hydrogen bond donors (HBD) and acceptors (HBA), and number of rotatable bonds (RB) was performed using SwissADME (http://swissadme.ch/index.php (accessed 16 February 2021)). 

## 3. Results and Discussion

### 3.1. Chemistry

**Scheme 1:**
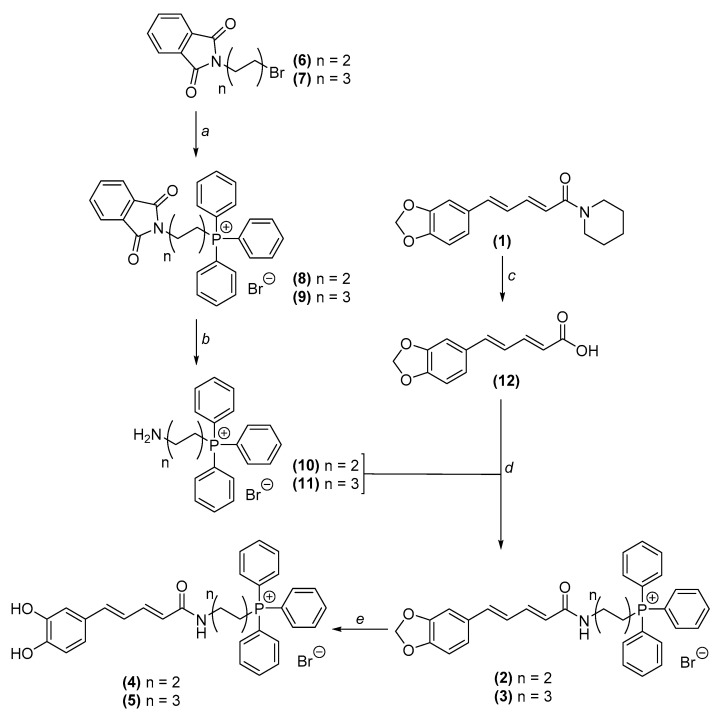
General procedure pursued to obtain the mitochondria-targeted compounds 2–5. (a) TPP, argon atmosphere, 130 °C, 1.5–4 h; (b) BuNH_2_, ethanol, reflux, 1.5–10 h; (c) Methanolic solution of NaOH 2 M, reflux, 24 h; (d) 1. dichloromethane, Et_3_N, ethyl chloroformate, rt, 2 h; 2. Compounds 10 and 11, rt, overnight; (e) BBr_3_.S(CH_3_)_2_, dichloromethane, argon atmosphere, reflux, 6 h.

The synthetic route used to obtain TPP^+^ conjugates 2–5 is depicted in Scheme 1. Aminoalkyltriphenylphosphonium salts 10 and 11 were prepared in 2 steps. The appropriate bromoalkylphthalymides (compounds 6 and 7) were heated with triphenylphosphine (TPP) to obtain compounds 8 and 9 (Scheme 1, step *a*), which in turn were refluxed with *n*-butylamine in ethanol to induce the phthalymidyl ring cleavage, yielding compounds 10 and 11 (Scheme 1, step *b*). Piperic acid (compound 12) was obtained by alkaline hydrolysis of piperine (compound 1) (Scheme 1, step *c*). Piperic acid was then acylated with ethyl chloroformate in alkaline media and reacted with amines 10 and 11 to afford compounds 2 and 3, respectively (Scheme 1, step *d*). Catechol derivatives 4 and 5 were prepared by the demethylenation of compounds 2 and 3, respectively, using boron tribromide dimethyl sulfide complex (BBr_3_.S(CH_3_)_2_). (Scheme 1, step *e*). The synthesis of AntiOXCIN2 and AntiOXCIN3 was performed as reported by Teixeira et al. [19].

### 3.2. Cholinesterase Inhibition Studies

#### 3.2.1. Evaluation of Electric Eel Acetylcholinesterase and Equine Butyrylcholinesterase Inhibitory Activities

The *ee*AChE and *eq*BChE inhibitory activities of piperine and derivatives thereof were evaluated using the Ellman’s method [32,33]. Donepezil, a reversible and selective AChE inhibitor commonly prescribed for AD [47,48], was used as a reference. The results are expressed as IC_50_ values and are displayed in Table 2. 

While piperine (1) did not display significant *ee*AChE and *eq*BChE inhibitory activities at 10 µM, compounds 2–5, AntiOXCIN2 and AntiOXCIN3 inhibited both *ee*AChE and *eq*BChE in our experimental conditions. Concerning *ee*AChE inhibition, the IC_50_ values were within the micromolar range. Compounds 4 and 5 displayed lower *ee*AChE IC_50_ values than AntiOXCIN2 and AntiOXCIN3, respectively, suggesting that the increased rigidification of the aliphatic chain improves *ee*AChE inhibition. Catechol derivatives **4** and **5** showed lower IC_50_ values than the counterparts containing the benzodioxole ring (compounds **2** and **3**, respectively). The spacer length (four or six carbon) did not significantly influence *ee*AChE inhibition. 

Remarkably, compounds **2**–**5**, AntiOXCIN2 and AntiOXCIN3 displayed higher potency towards *eq*BChE, presenting IC_50_ values within the nanomolar range. As observed in *ee*AChE inhibition, catechol derivatives with conjugated double bonds (compounds **4** and **5**) exhibited lower *eq*BChE than the related cinnamoyl counterparts (AntiOXCIN2 and AntiOXCIN3, respectively). Moreover, the derivatives bearing a six-carbon chain (compounds 3 and 5) were better *eq*BChE inhibitors than the four-carbon chain analogues (compounds 2 and 4). In contrast to the results obtained with *ee*AChE, benzodioxole derivatives 2 and 3 showed lower IC_50_ values than catechols 4 and 5, respectively. 

The *eq*BChE selectivity index, determined from the ratio of the IC_50_ values of *ee*AChE and *eq*BChE, showed that benzodioxole derivatives 2 and 3 were more selective towards *eq*BChE (226-fold and 314-fold, respectively) than catechols 4 and 5 (36-fold and 62-fold, respectively). In addition, compounds **4**, **5**, AntiOXCIN2 and AntiOXCIN3 presented *eq*BChE selectivity index within the same range, indicating that the selectivity towards *eq*BChE was maintained with the increased rigidification of the aliphatic chain.

#### 3.2.2. Evaluation of Human Acetylcholinesterase and Butyrylcholinesterase Inhibitory Activities

Based on the promising results obtained in *ee*AChE and *eq*BChE inhibition studies, compounds **2**–**5** were selected to further investigate their inhibitory activities towards human ChEs (*h*ChEs). The data obtained are presented in Table 2. 

Compared with the results obtained with *ee*AChE and *eq*BChE, differences in the inhibition potency and selectivity of compounds **2**–**5** towards *h*ChEs were observed. In general, TPP^+^ conjugates **2**–**5** were less active towards *h*ChEs, with the differences being more pronounced between the data obtained with *h*BChE and *eq*BChE. The lower potency of several compounds towards *h*ChEs was also observed in other studies [49,50]. Although *ee*AChE and *eq*BChE share > 88 % of amino acid identity with the human ChEs [50,51], they may present distinct structural and dynamic features. For instance, *eq*BChE and *h*BChE present different volume of the active site and exhibit three specific residue variations in the gorge site (Val305, Asp311 and Leu313 in *eq*BChE are replaced with Ala277, Gly283 and Pro285 in *h*BChE, respectively) [50]. These differences may invariably influence the binding of compounds to the enzymes and reflect in the ChE inhibitory activities.

All TPP^+^ conjugates presented IC_50_ values towards *h*AChE and *h*BChE within the low micromolar or high nanomolar range. While compounds containing a four-carbon chain were more selective towards *h*BChE (compound 2: SI = 55; compound 4: SI = 7.3), compounds bearing a six-carbon chain exhibited higher selectivity for *h*AChE (compound 3: SI = 0.67; compound 5: SI = 0.39). Interestingly, compounds containing six-carbon alkyl chains (compounds **3** and **5**) displayed lower *h*AChE IC_50_ values and higher *h*BChE IC_50_ values than the four-carbon chain counterparts (compounds **2** and **4**, respectively). Indeed, compound **3** was the most potent *h*AChE inhibitor of the series (compound **3**: *h*AChE = 2 µM), while compound **2** presented the lowest IC_50_ towards *h*BChE (compound **2**: *h*AChE = 200 nM). Moreover, benzodioxole derivatives **2** and **3** were more potent *h*AChE and *h*BChE inhibitors than catechol derivatives **4** and **5**, respectively. Overall, these results suggest different binding modality and interaction of TPP^+^ conjugates towards *h*AChE and *h*BChE. 

#### 3.2.3. Crystallographic Studies with Human Cholinesterases

**Figure 2:**
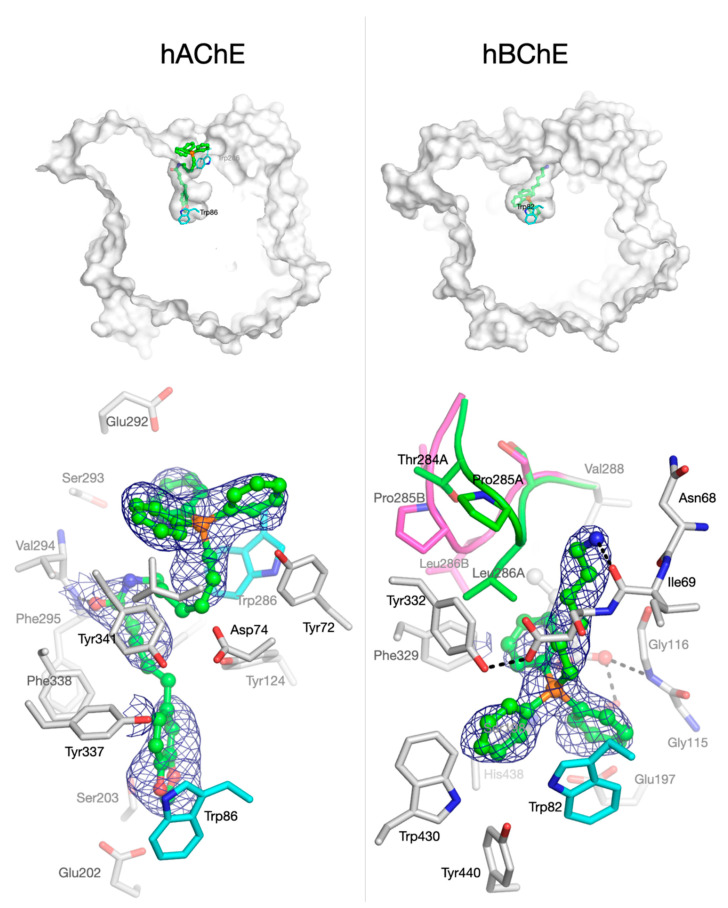
Views of compound **3** in complex with *h*AChE (PDB 6ZWE) and *h*BChE (PDB 6ZWI). Compound **3** is shown in green ball and sticks. Top: Overview of compound **3** location inside the gorge of *h*ChEs defined by the solvent accessible surface, showing the TPP^+^ moiety outside the gorge of *h*AChE (left) and inside and at the bottom of the gorge of *h*BChE (right). Bottom: Closer view of compound **3** inside the gorge of *h*AChE and *h*BChE. A 1-σ feature-enhanced map is represented as a blue mesh [52]. Residues lining the gorge are represented in sticks with carbon in white. Key peripheral (Trp286) and active site tryptophan residues (Trp86 and Trp82) are represented in sticks with carbon in cyan. The two alternate conformations of the acyl-loop of *h*BChE are represented in stick and cartoon, with carbons in green (conformation A) and magenta (conformation B).

To gain insight into the binding mode of TPP^+^ conjugates to *h*AChE and *h*BChE, attempts to solve the crystallographic structures of the complexes formed between compounds **2**–**5** and recombinant *h*ChEs were conducted. Structures of the complexes formed between compounds **2**–**5** and *h*AChE were solved (data not shown). Structures of *h*BChE in complex with compounds **3** and **5** were also obtained (data not shown). Compounds **2**–**5** bind similarly to *h*AChE and compounds **3** and **5** bind similarly to *h*BChE, although distinct binding modes of compounds to *h*AChE and *h*BChE were observed. Figure 2 shows an overview of the binding of compound **3** to *h*AChE and *h*BChE (Top) and a corresponding closer view of the molecule inside the active site of each enzyme (Bottom). 

For *h*AChE, the electron density map corresponding to compound 3 can be observed in the active site of the enzyme, identifying clearly the location of TPP^+^ moiety at the entrance of the active-site gorge of the enzyme (Figure 2, bottom left). Strong stacking interaction between aromatic amino acids located at the rim of the gorge of *h*AChE and the TPP^+^ moiety was not detected. The electron density map locates the benzodioxole moiety of compound **3** at the bottom of the gorge in a pseudo T-staking interaction with Trp86. Parts of the electron density of the linker between the TPP^+^ moiety and the benzodioxole ring of compound **3** are missing. Nevertheless, electron density is clearly present around the oxygen atom of the amide function interacting with Phe295. 

Compound **3** appears to bind *h*BChE in an opposite way than *h*AChE: the electron density map of compound 3 clearly indicates the location of the TPP^+^ moiety at the bottom of *h*BChE active site (Figure 1, bottom right). The wider opening and overall volume of *h*BChE active site (500 Å^3^), compared to *h*AChE (300 Å^3^) [53], allows the TPP^+^ moiety to enter the gorge and bind in the vicinity of the catalytic site. A π-stacking interaction between Trp82 and two phenyl groups of the TPP^+^ moiety may stabilize the molecule inside the active site. It is important to note that, in previous studies of our research group, both the fitting orientation of TPP^+^ compounds within the active site gorge of *h*BChE and their interactions with Trp82 were predicted in docking simulations [17,22]. Missing electron density of compound **3** indicates that the piperoyl moiety is not visible. This moiety may be freely spanning outside the active site of *h*BChE. The binding of compound 3 to *h*BChE induces a position shift of Phe329 compared to 1P0I, but the most striking result is the coexistence of alternate conformations of the acyl-binding loop (Thr284-Pro285-Leu 286; Figure 2, bottom right). Such an extensive rearrangement of the acyl-loop has never been observed before for *h*BChE. In addition, as usually seen in the X-ray structures of this enzyme, an unidentified 5-carbon long carboxylic acid is bound in the vicinity of the catalytic serine extending its alkyl chain in the acyl-binding pocket.

Thus, the data obtained from the resolved crystal complex of compound **3** with *h*BChE is highly relevant for two main reasons. First, this is the first time the binding mode of TPP^+^ conjugates with *h*BChE is experimentally demonstrated; second, the data constitutes per se a validation of our molecular docking data obtained in previous studies [17,22].

### 3.3. Monoamine Oxidase Inhibition Studies

We evaluated the *h*MAO inhibition properties of piperine (compound 1) and TPP^+^ conjugates **2**–**5**, using kynuramine as substrate and recombinant *h*MAO-A and -B isoforms [29,40]. The *h*MAO-A and *h*MAO-B inhibitory potency (IC_50_) and selectivity (SI) data of the compounds under study and reference inhibitors (clorgyline for *h*MAO-A and (R)-(−)-deprenyl, rasagiline, safinamide for *h*MAO-B) are reported in Table 3.

In our previous reports, piperine showed moderate and selective *h*MAO-B inhibitory activity (compound 1, IC_50_ = 1.05 µM, SI = 10) [29]. AntiOXCIN2 and AntiOXCIN3 did not significantly inhibit *h*MAOs at 10 µM (% inhibition < 50 %). In contrast, compounds 2–5 inhibited both *h*MAO isoforms, presenting IC_50_ values within the micromolar or high nanomolar range. The differences in the compounds’ rigidity and/or in the position of the carbonyl group may be underlying the different *h*MAO inhibition profiles of compounds 2–5 and AntiOXCINs. Compounds 2 and 3 displayed similar or lower IC_50_ values towards *h*MAOs than compounds 4 and 5, respectively. Therefore, the benzodioxole ring opening had a negative effect on the ability to inhibit both *h*MAO isoforms. Concerning the spacer length, compounds containing a six-carbon chain (compounds 3 and 5) were more effective *h*MAO-B inhibitors than compounds with a four-carbon chain (compounds 2 and 4). However, no correlation was found between *h*MAO-A inhibition and spacer length.

### 3.4. Antioxidant Activity

To evaluate the radical scavenging activity of piperine (compound 1), compounds 2–5, AntiOXCIN2 and AntiOXCIN3, we used the oxygen radical absorbance capacity (ORAC) assay. This method measures the antioxidant activity against peroxyl radical (ROO•)-induced oxidation of fluorescein, a fluorescent probe, into a non-fluorescent product [54]. While piperine (compound 1) and compounds 2 and 3 were unable to scavenge ROO• radicals, catechols 4 and 5, AntiOXCIN2 and AntiOXCIN3 effectively protected fluorescein from oxidation (Table 4). Compounds 4 and 5 displayed lower ORAC-FL indexes than AntiOXCIN2 and AntiOXCIN3, respectively. Thus, the π-system extension in the chemical structure of AntiOXCINs enhanced the compounds’ ability to act as chain-breaking antioxidants against ROO• radicals.

### 3.5. Electrochemical Studies

**Figure 3:**
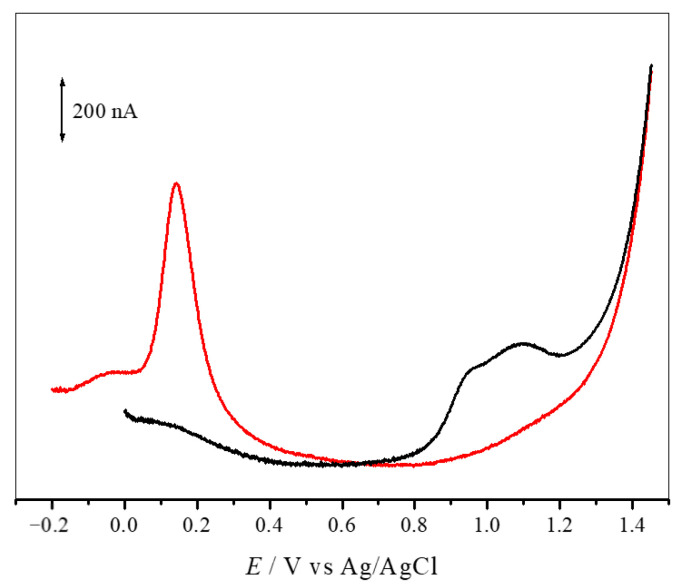
Differential pulse voltammogram for 0.1 mM solutions of (▬) compound 3 and (▬) compound 5 in physiological pH 7.4 supporting electrolyte. Scan rate: 5 mV·s^−1^.

The redox behaviour of compounds **2**–**5** and AntiOXCINs was studied by differential pulse voltammetry (DPV) at physiological pH 7.4, using a glassy carbon-working electrode. Benzodioxole derivatives 2 and 3 presented two overlapped anodic peaks at physiological pH. The high values of redox potential (*E*_p_) values obtained for these compounds are consistent with molecules that exhibit poor antioxidant activity. Indeed, compounds **2** and **3** do not present aromatic hydroxyl groups that can be oxidized, being the conjugated dienone system the most probable site for oxidation [55]. In contrast, catechols 4 and 5 displayed well-defined anodic peaks associated with the oxidation of the catechol groups (Figure 3). The redox potential (*E*_p_) values obtained for compounds 4 and 5 were 125 mV and 144 mV (Table 4), respectively. The *E*_p_ values of compounds 4 and 5 were lower than those obtained with AntiOXCIN2 (*E*_p_ = 166 mV) and AntiOXCIN3 (*E*_p_ = 164 mV) [19]. Therefore, the higher electron delocalization between the aromatic ring and the carbonyl group shifted the peak potentials towards less positive values, thus enhancing the reducing power of catechols.

### 3.6. Evaluation of Cytotoxicity Profile

**Figure 4:**
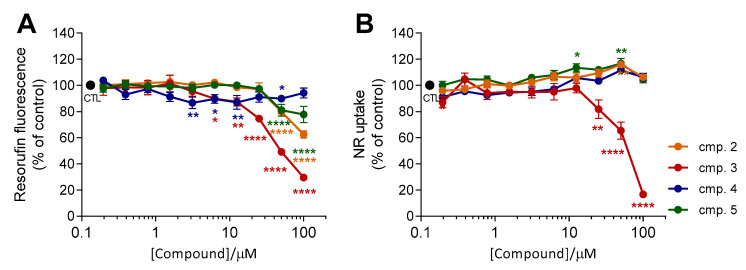
Cellular viability of differentiated SH-SY5Y neuroblastoma cells after a 24 h treatment with increasing concentrations of compounds 2–5 (0.20–100 µM). Cellular viability was evaluated using the resazurin reduction assay (**A**) and the NR uptake assay (**B**). Results are expressed as the mean % of untreated controls ± SEM (*n* = 3). Statistical comparisons were made using two-way ANOVA followed by Dunnet’s multiple comparison test. In all cases, *p* values lower than 0.05 were considered significant (* *p* < 0.05, ** *p* < 0.01, **** *p* < 0.0001 vs untreated cells).

To evaluate the cytotoxicity of TPP^+^ conjugates 2–5, differentiated SH-SY5Y cells were incubated with increasing concentrations of the test compounds (0.20–100 µM) for 24 h. Cellular cytotoxicity was assessed using the resazurin reduction assay, which estimates metabolic activity of viable cells [56], and the neutral red (NR) uptake assay, which relies on the lysosomal incorporation of the dye NR in living cells [57]. The results, presented as mean resorufin fluorescence (% of control) ± SEM and NR uptake (% of control) ± SEM (*n* = 3), are depicted in Figure 4.

Data obtained in our previous reports showed that piperine (compound 1) did not exhibit cytotoxic effects at concentrations up to 50 µM [58]. Similarly, AntiOXCIN2 and AntiOXCIN3 did not markedly affect both resazurin reduction and NR uptake at concentrations up to 100 µM (Figure S1). However, a significant dose-dependent decrease in resorufin fluorescence was observed with compounds 2, 3, and 5 (Figure 4A). Compound 3 presented the most significant decreases in resazurin reduction (resorufin fluorescence < 85%) at concentrations above 12.5 µM (Figure 4A). This tendency was also observed with compounds **2** and **5** at concentrations above 25 µM (Figure 4A). On the other hand, incubation of neuroblastoma cells with increasing concentrations of compound 4 up to 100 µM did not markedly affect resazurin reduction (Figure 4A). 

Resazurin reduction was influenced by the substitution pattern of the aromatic ring and by the length of the lipophilic spacer. The effects of the structural components of TPP^+^ cations **2**–**5** were particularly noticeable at the highest tested concentration (100 µM). In fact, decreases of resazurin reduction were more prominent by treatment with benzodioxoles (compounds **2** and **3**) than by catechols (compounds **4** and **5**) (Figure 4A). Moreover, compounds bearing a four-carbon alkyl chain (compounds **2** and **4**) led to a lower decrease in resazurin reduction than the six-carbon alkyl chain analogues (compounds **3** and **5**, respectively) (Figure 4A).

Interestingly, the same conclusions were not similar when using the NR uptake assay (Figure 4B). Neutral red (NR) uptake remained constant with increasing concentrations of compounds **2**, **4** and **5** (Figure 4B), even at concentrations in which the resazurin reduction was significantly decreased (Figure 4A, compounds **2** and **5**, ≥ 50 µM). Only compound **3** displayed the same tendency observed with the resazurin reduction assay, showing significant lysosomal toxicity at concentrations above 12.5 µM. 

In summary, these results indicate that catechol TPP^+^ conjugates are less prone to decrease cellular metabolism at high concentrations than the corresponding benzodioxole derivatives. Moreover, catechols 4 and 5 share the same cytotoxicity profile of the parent AntiOXCINs. 

### 3.7. Evaluation of Drug-Like Properties

To study the drug-likeness of TPP^+^ conjugates 2–5, AntiOXCIN2 and AntiOXCIN3, we first determined their chromatographic hydrophobic index (CHI) LogP_oct_ (CHI LogP_oct_). The CHI parameter is derived from the retention times obtained in a fast gradient reversed-phase HPLC method [59]. Chromatographic hydrophobicity index (CHI) values of neutral molecules are directly correlated with the compounds’ lipophilicity [59]. In our case, the determination of CHI was carried out under acidic conditions (pH 2.3) to prevent the ionization of the catechol moiety. The values of CHI were then used to calculate CHI LogP_oct_. The results are presented in Table 5.

Chromatographic hydrophobicity index LogP_oct_ (CHI LogP_oct_) values of compounds **2**–**5** were considerably lower than the obtained with piperine (Table 5). Therefore, despite the high lipophilicity of the TPP^+^ moiety, the positive charge of TPP^+^ conjugates may decrease the affinity towards the hydrophobic stationary phase. Moreover, CHI LogP_oct_ values increased with the presence of a benzodioxole ring (compounds 2 and 3 vs compounds **4** and **5**, respectively) and longer alkyl linkers (compounds **2** and **4** vs. compounds **3** and **5**, respectively) (Table 4). Finally, CHI LogP_oct_ values of compounds **4** and **5** were similar to those of AntiOXCIN2 and AntiOXCIN3, respectively, suggesting that the π-system extension maintained the compounds’ hydrophobicity.

We also calculated several physicochemical properties to predict the ability of compounds **2**–**5**, AntiOXCIN2 and AntiOXCIN3 to attain the CNS. These include molecular weight (MW), topological polar surface area (TPSA in Å), number of hydrogen acceptors (HBA), number of hydrogen donors (HBD) and number of rotatable bonds (RB) (Table 5). Among the estimated parameters, MW and RB values of TPP^+^ conjugates exceeded the limits suggested for CNS-active drugs (MW < 500 g·mol^−1^; RB < 8). Nonetheless, despite their high volume, several lipophilic TPP^+^ cations were taken up by mitochondria within several tissues (brain, heart, liver) following long-term oral administration [63]. In addition, our previous studies showed that catechol and pyrogallol lipophilic TPP^+^ conjugates can cross human cerebral microvascular endothelial (*h*CMEC/D3) cells, an in vitro cellular model of human BBB, in a dose-dependent manner [21]. As expected, compounds **4** and **5** presented a lower number of RB than AntiOXCIN2 and AntiOXCIN3, respectively. The other predicted physicochemical properties (TPSA, HBA, HBD) fell within the proposed limits. 

## 4. Conclusions

In this work, we compared the bioactivity and toxicity profiles of two AntiOXCINs with bioisosteres with extended electron delocalization and similar lipophilicity. The increased rigidity of lipophilic TPP^+^ conjugates enhanced *ee*AChE and *eq*BChE inhibition while preserving the selectivity index towards *eq*BChE. Co-crystallization studies with *h*ChEs demonstrated that the TPP^+^ derivatives bind differently to the active sites of *h*AChE and *h*BChE. While the TPP^+^ moiety was present at the entrance of the active-site gorge of *h*AChE, in *h*BChE it was located within the gorge in the vicinity of the active site. These data validated our results from docking simulations performed for similar derivatives that predicted the binding mode of lipophilic TPP^+^ conjugates to the active site of *h*BChE [17,22]. Unlike AntiOXCINs, compounds 2–5 are endowed with moderate *h*MAO inhibition properties. In addition, catechols **4** and **5** scavenged ROO• radicals more efficiently and presented lower *E*_p_ values than AntiOXCIN2 and AntiOXCIN3, respectively. Cytotoxicity studies showed that catechols compounds **4** and **5** are less cytotoxic than the related benzodioxole-containing compounds (compounds **2** and **3**). Moreover, the safety profile of compounds 4 and 5 at concentrations up to 100 µM was similar to that of AntiOXCIN2 and AntiOXCIN3, respectively. 

Compound **4** stands out as the best mitochondria-targeted agent of the series. In addition to the dual ChE/MAO inhibition profile, compound 4 is endowed with antioxidant activity. Compound **4** also presents a safe cytotoxicity profile in differentiated neuroblastoma cells (<100 µM) and favourable drug-like properties.

Overall, this work represents an advance towards the development of mitochondria-targeted antioxidants with multitarget activity. These results can potentially propel the discovery of new compounds able to tackle simultaneously neurotransmitter depletion and mitochondria-associated oxidative stress in NDs.

## Figures and Tables

**Table 1:** Data collection and refinement statistics. Calculated using Phenix [39]. R-work=Σ |fo − |fc||/Σ |fo|, fo and fc are observed and calculated structure factors, R-free set uses about thousands randomly chosen reflections. Statistics for the highest-resolution shell are shown in parentheses.

PDB Entry Code	6ZWE	6ZWI
X-ray source	PROXIMA 2	PROXIMA 2
Wavelenght	0.9801	0.9801
Resolution range	78.09–3.0 (3.107–3.0)	41.48–1.85 (1.916–1.85)
Space group	P 61	I 4 2 2
Unit cell	a = 211.35, b = 211.35, c = 115.9 α = β = 90.0, γ = 120	a = 154.3, b = 154.3, c = 127.7 α = β = γ = 90.0
Total reflections	2352044 (194838)	3366060 (268864)
Unique reflections	59080 (5885)	65496 (6485)
Multiplicity	39.8 (33.1)	51.4 (41.5)
Completeness (%)	99.87 (99.66)	99.96 (99.92)
Mean I/sigma(I)	20.81 (2.80)	29.97 (0.81)
Wilson B-factor	85.22	47.10
R-merge	0.1637 (1.526)	0.1014 (6.341)
R-meas	0.1658 (1.55)	0.1025 (6.418)
R-pim	0.02615 (0.2668)	0.01425 (0.9876)
CC1/2	0.999 (0.933)	1 (0.31)
CC *	1 (0.982)	1 (0.688)
Reflections used in refinement	59025 (5879)	65488 (6482)
Reflections used for R-free	1180 (118)	1309 (129)
R-work	0.1982 (0.3401)	0.1878 (0.3676)
R-free	0.2411 (0.3895)	0.2241 (0.3844)
Number of non-hydrogen atoms	8782	4808
macromolecules	8363	4294
ligands	274	286
solvent	145	228
Protein residues	1073	527
RMS(bonds)	0.004	0.016
RMS(angles)	0.88	1.27
Ramachandran favored (%)	95.03	95.62
Ramachandran allowed (%)	4.87	4.19
Ramachandran outliers (%)	0.09	0.19
Rotamer outliers (%)	0.11	0.43
Clashscore	11.09	5.34
Average B-factor	97.42	56.31
macromolecules	96.06	53.61
ligands	143.73	96.23
solvent	88.44	57.18

**Table 2:** Evaluation of ChE (*ee*AChE, *eq*BChE, *h*AChE, *h*BChE) inhibitory activity of piperine (compound 1), compounds 2–5, AntiOXCIN2 and AntiOXCIN3 and reference inhibitor.

Compound	IC_50_/µM		SI (1) *^b^*	IC_50_/µM		SI (2) *^c^*
	*ee*AChE	*eq*BChE		*h*AChE	*h*BChE	
1	___ *^a^*	___*^a^*	___	___ *^e^*	___ *^e^*	___
2	6.39 ± 0.28	0.0282 ± 0.0014	226	11 ± 1	0.20 ± 0.04	55
3	5.74 ± 0.32	0.0179 ± 0.0009	314	2.0 ± 0.2	3.0 ± 0.4	0.67
4	2.23 ± 0.12	0.0619 ± 0.0042	36	22 ± 2	3.0 ± 0.2	7.3
5	2.14 ± 0.06	0.0341 ± 0.0029	62	9.0 ± 0.5	23 ± 2	0.39
AntiOXCIN2	6.32 ± 0.14 *^d^*	0.124 ± 0.007 *^d^*	51 *^d^*	___ *^e^*	___ *^e^*	___
AntiOXCIN3	5.08 ± 0.22	0.325 ± 0.09	16	___ *^e^*	___ *^e^*	___
Donepezil	0.0129 ± 0.0008	2.50 ± 0.09	0.0052	___ *^e^*	___ *^e^*	___

*^a^* Compounds did not reach 50% inhibition at the highest compound concentration tested (10 µM); *^b^* SI (1): *eq*BChE selectivity index = IC_50_(*ee*AChE)/IC_50_(*eq*BChE). *^c^* SI (2): *h*BChE selectivity index = IC_50_(*h*AChE)/IC_50_(*h*BChE). *^d^* Data from Benfeito et al. [22]. *^e^* Not determined.

**Table 3:** Evaluation of *h*MAOs (*h*MAO-A and *h*MAO-B) inhibitory activity of piperine (compound 1), compounds 2–5, AntiOXCIN2, AntiOXCIN3 and reference inhibitors.

Compound	IC_50_/µM		SI (3) *^b^*
	*h*MAO-A	*h*MAO-B	
1	___ *^a,d^*	1.05 ± 0.08 *^d^*	>10 *^c,^^d^*
2	0.888 ± 0.022	12.4 ± 1.9	0.07
3	1.23 ± 0.13	4.64 ± 0.29	0.26
4	5.17 ± 0.54	13.5 ± 1.1	0.38
5	2.17 ± 0.28	10.9 ± 0.7	0.20
AntiOXCIN2	___ *^a^*	___ *^a^*	__
AntiOXCIN3	___ *^a^*	___ *^a^*	__
R-(−)-Deprenyl	20.1 ± 1.9	0.0386 ± 0.0043	522
Rasagiline	3.65 ± 0.31	147.3 ± 249	24
Safinamide	___ *^a^*	0.0231 ± 0.0026	>433 *^c^*
Clorgyline	0.00274 ± 0.00047	2.21 ± 0.26	0.00124

*^a^* Compounds did not reach 50% inhibition at the highest compound concentration tested (10 µM); *^b^* SI (3): *h*MAO-B selectivity index = IC_50_ (*h*MAO-A)/IC_50_ (*h*MAO-B). *^c^* Values obtained under the assumption that the corresponding IC_50_ against *h*MAO-A or *h*MAO-B is the highest concentration tested (10 μM). *^d^* Data from Chavarria et al. [29].

**Table 4:** ORAC-FL indexes and redox potentials (*E*_p_) of piperine (compound 1), compounds 2–5, AntiOXCIN2 and AntiOXCIN3.

Compound	ORAC-FL Index	*E*_p_/mV
1	___	n.d.
2	___	942; 1070
3	___	931; 1083
4	3.3 ± 0.1	125
5	3.1 ± 0.3	144
AntiOXCIN2	2.8 ± 0.1	166 *^a^*
AntiOXCIN3	2.6 ± 0.1	164 *^a^*

*^a^* Data from Teixeira et al. [19,20]. n.d.: not determined.

**Table 5:** Calculated CHI and CHI Log P_oct_ and predicted drug-like properties of piperine (compound 1) and TPP^+^ conjugates 2–5, AntiOXCIN2 and AntiOXCIN3.

Compound	CHI *^a^*	CHI LogP_oct_ *^b^*	MW *^c^*	TPSA *^c^*	HBA *^c^*	HBD *^c^*	RB *^c^*
1	73.4	2.35	285.3	38.77	3	0	4
2	42.8	1.27	614.5	61.15	4	1	12
3	45.8	1.41	642.6	61.15	4	1	14
4	34.4	1.59	602.5	83.15	4	3	12
5	37.2	1.73	630.6	83.15	4	3	14
AntiOXCIN2	37.9	1.76	619.7	83.15	4	3	14
AntiOXCIN3	41.4	1.92	647.8	83.15	4	3	16
CNS^+^ drugs	___	___	<500 [60]	<90 [60]	<7 [61]	<3 [61]	<8 [61]

*^a^* CHI values were calculated using the equation obtained in the linear correlation (see supplementary information, Figure S2). *^b^* CHI logP values were back-calculated using the equation ${\mathrm{CHI}\;\mathrm{LogP}}_{\mathrm{oct}}\;=\;0.047\;\times \;\mathrm{CHI}\;+\;0.36\;\times \;\mathrm{HBC}\;-\;1.10$ [62]. *^c^* Properties predicted using SwissADME (http://swissadme.ch/index.php (accessed on 7 December 2020)). MW: molecular weight; TPSA: topological polar surface area; HBA: number of H-bond acceptor atoms; HBD: number of H-bond donor atoms; RB: number of rotatable bonds.

## Data Availability

The data presented in this study are available within the article and its supplementary material. Other data that support the findings of this study are available upon request from the corresponding authors.

## Supplementary Materials

The following are available online: https://www.mdpi.com/2076-3921/10/2/329/s1. Experimental details, Tables, Figures. Table S1. Retention times (t_R_) of the standard mixture obtained by LC/UV at pH 2.3. Figure S1. Cellular viability of differentiated SH-SY5Y neuroblastoma cells after a 24 h treatment with AntiOXCIN2 and AntiOXCIN3 at eight different concentrations (0.20–100 µM). Figure S2. Linear correlation obtained by plotting the retention times (t_R_) of each of the individual standard mixture compounds against the CHI values at pH 2.3 (CHI_0_ pH 2.3). Figure S3. ^1^H and ^13^C NMR spectra of compound 2 (NMR spectra obtained in CDCl_3_-*d*_1_). Figure S4. ^1^H and ^13^C NMR spectra of compound 3 (NMR spectra obtained in CDCl_3_-*d*_1_). Figure S5. ^1^H and ^13^C NMR spectra of compound 4 (NMR spectra obtained in DMSO-*d*_6_). Figure S6. ^1^H and ^13^C NMR spectra of compound 5 (NMR spectra obtained in DMSO-*d*_6_).

## References

[1] Ramsay R.R., Majekova M., Medina M., Valoti M. (2016). Key Targets for Multi-Target Ligands Designed to Combat Neurodegeneration. Front. Neurosci..

[2] Guzior N., Wieckowska A., Panek D., Malawska B. (2015). Recent development of multifunctional agents as potential drug candidates for the treatment of Alzheimer’s disease. Curr. Med. Chem..

[3] Elmabruk A., Das B., Yedlapudi D., Xu L., Antonio T., Reith M.E.A., Dutta A.K. (2019). Design, Synthesis, and Pharmacological Characterization of Carbazole Based Dopamine Agonists as Potential Symptomatic and Neuroprotective Therapeutic Agents for Parkinson’s Disease. ACS Chem. Neurosci..

[4] Rosini M., Simoni E., Milelli A., Minarini A., Melchiorre C. (2014). Oxidative stress in Alzheimer’s disease: Are we connecting the dots?. J. Med. Chem..

[5] Fetisova E.K., Avetisyan A.V., Izyumov D.S., Korotetskaya M.V., Chernyak B.V., Skulachev V.P. (2010). Mitochondria-targeted antioxidant SkQR1 selectively protects MDR (Pgp 170)-negative cells against oxidative stress. FEBS Lett..

[6] Johri A., Beal M.F. (2012). Mitochondrial dysfunction in neurodegenerative diseases. J. Pharmacol. Exp. Ther..

[7] Lezi E., Swerdlow R.H. (2012). Mitochondria in neurodegeneration. Adv. Exp. Med. Biol..

[8] Golpich M., Amini E., Mohamed Z., Azman Ali R., Mohamed Ibrahim N., Ahmadiani A. (2017). Mitochondrial Dysfunction and Biogenesis in Neurodegenerative diseases: Pathogenesis and Treatment. CNS Neurosci. Ther..

[9] Facecchia K., Fochesato L.A., Ray S.D., Stohs S.J., Pandey S. (2011). Oxidative toxicity in neurodegenerative diseases: Role of mitochondrial dysfunction and therapeutic strategies. J. Toxicol..

[10] Jellinger K.A. (2010). Basic mechanisms of neurodegeneration: A critical update. J. Cell. Mol. Med..

[11] Biasutto L., Mattarei A., La Spina M., Azzolini M., Parrasia S., Szabò I., Zoratti M. (2019). Strategies to target bioactive molecules to subcellular compartments. Focus on natural compounds. Eur. J. Med. Chem..

[12] Milane L., Trivedi M., Singh A., Talekar M., Amiji M. (2015). Mitochondrial biology, targets, and drug delivery. J. Control. Release.

[13] Guzman-Villanueva D., Weissig V. (2017). Mitochondria-Targeted Agents: Mitochondriotropics, Mitochondriotoxics, and Mitocans. Handb. Exp. Pharmacol..

[14] He H., Li D.W., Yang L.Y., Fu L., Zhu X.J., Wong W.K., Jiang F.L., Liu Y. (2015). A novel bifunctional mitochondria-targeted anticancer agent with high selectivity for cancer cells. Sci. Rep..

[15] Zielonka J., Joseph J., Sikora A., Hardy M., Ouari O., Vasquez-Vivar J., Cheng G., Lopez M., Kalyanaraman B. (2017). Mitochondria-Targeted Triphenylphosphonium-Based Compounds: Syntheses, Mechanisms of Action, and Therapeutic and Diagnostic Applications. Chem. Rev..

[16] Porteous C.M., Logan A., Evans C., Ledgerwood E.C., Menon D.K., Aigbirhio F., Smith R.A., Murphy M.P. (2010). Rapid uptake of lipophilic triphenylphosphonium cations by mitochondria in vivo following intravenous injection: Implications for mitochondria-specific therapies and probes. Biochim. Biophys. Acta.

[17] Oliveira C., Cagide F., Teixeira J., Amorim R., Sequeira L., Mesiti F., Silva T., Garrido J., Remiao F., Vilar S. (2018). Hydroxybenzoic Acid Derivatives as Dual-Target Ligands: Mitochondriotropic Antioxidants and Cholinesterase Inhibitors. Front. Chem..

[18] Teixeira J., Oliveira C., Amorim R., Cagide F., Garrido J., Ribeiro J.A., Pereira C.M., Silva A.F., Andrade P.B., Oliveira P.J. (2017). Development of hydroxybenzoic-based platforms as a solution to deliver dietary antioxidants to mitochondria. Sci. Rep..

[19] Teixeira J., Cagide F., Benfeito S., Soares P., Garrido J., Baldeiras I., Ribeiro J.A., Pereira C.M., Silva A.F., Andrade P.B. (2017). Development of a Mitochondriotropic Antioxidant Based on Caffeic Acid: Proof of Concept on Cellular and Mitochondrial Oxidative Stress Models. J. Med. Chem..

[20] Teixeira J., Soares P., Benfeito S., Gaspar A., Garrido J., Murphy M.P., Borges F. (2012). Rational discovery and development of a mitochondria-targeted antioxidant based on cinnamic acid scaffold. Free Radic. Res..

[21] Benfeito S., Oliveira C., Fernandes C., Cagide F., Teixeira J., Amorim R., Garrido J., Martins C., Sarmento B., Silva R. (2019). Fine-tuning the neuroprotective and blood-brain barrier permeability profile of multi-target agents designed to prevent progressive mitochondrial dysfunction. Eur. J. Med. Chem..

[22] Benfeito S., Fernandes C., Vilar S., Remião F., Uriarte E., Borges F. (2020). Exploring the multi-target performance of mitochondriotropic antioxidants against the pivotal Alzheimer’s disease pathophysiological hallmarks. Molecules.

[23] Yang W., Chen Y.H., Liu H., Qu H.D. (2015). Neuroprotective effects of piperine on the 1-methyl-4-phenyl-1,2,3,6-tetrahydropyridine-induced Parkinson’s disease mouse model. Int. J. Mol. Med..

[24] Wattanathorn J., Chonpathompikunlert P., Muchimapura S., Priprem A., Tankamnerdthai O. (2008). Piperine, the potential functional food for mood and cognitive disorders. Food Chem. Toxicol..

[25] Chonpathompikunlert P., Wattanathorn J., Muchimapura S. (2010). Piperine, the main alkaloid of Thai black pepper, protects against neurodegeneration and cognitive impairment in animal model of cognitive deficit like condition of Alzheimer’s disease. Food Chem. Toxicol..

[26] Liu J., Chen M., Wang X., Wang Y., Duan C., Gao G., Lu L., Wu X., Wang X., Yang H. (2016). Piperine induces autophagy by enhancing protein phosphotase 2A activity in a rotenone-induced Parkinson’s disease model. Oncotarget.

[27] Mao Q.Q., Huang Z., Ip S.P., Xian Y.F., Che C.T. (2012). Protective effects of piperine against corticosterone-induced neurotoxicity in PC12 cells. Cell. Mol. Neurobiol..

[28] Lee S.A., Hong S.S., Han X.H., Hwang J.S., Oh G.J., Lee K.S., Lee M.K., Hwang B.Y., Ro J.S. (2005). Piperine from the fruits of Piper longum with inhibitory effect on monoamine oxidase and antidepressant-like activity. Chem. Pharm. Bull..

[29] Chavarria D., Cagide F., Pinto M., Gomes L.R., Low J.N., Borges F. (2019). Development of piperic acid-based monoamine oxidase inhibitors: Synthesis, structural characterization and biological evaluation. J. Mol. Struct..

[30] Cheng G., Zielonka J., Ouari O., Lopez M., McAllister D., Boyle K., Barrios C.S., Weber J.J., Johnson B.D., Hardy M. (2016). Mitochondria-Targeted Analogues of Metformin Exhibit Enhanced Antiproliferative and Radiosensitizing Effects in Pancreatic Cancer Cells. Cancer Res..

[31] Williard P.G., Fryhle C.B. (1980). Boron trihalide-methyl sulfide complexes as convenient reagents for dealkylation of aryl ethers. Tetrahedron Lett..

[32] Ellman G.L., Courtney K.D., Andres V., Feather-Stone R.M. (1961). A new and rapid colorimetric determination of acetylcholinesterase activity. Biochem. Pharmacol..

[33] Di Giovanni S., Borloz A., Urbain A., Marston A., Hostettmann K., Carrupt P.A., Reist M. (2008). In vitro screening assays to identify natural or synthetic acetylcholinesterase inhibitors: Thin layer chromatography versus microplate methods. Eur. J. Pharm. Sci..

[34] Zueva I., Dias J., Lushchekina S., Semenov V., Mukhamedyarov M., Pashirova T., Babaev V., Nachon F., Petrova N., Nurullin L. (2019). New evidence for dual binding site inhibitors of acetylcholinesterase as improved drugs for treatment of Alzheimer’s disease. Neuropharmacology.

[35] Brazzolotto X., Wandhammer M., Ronco C., Trovaslet M., Jean L., Lockridge O., Renard P.Y., Nachon F. (2012). Human butyrylcholinesterase produced in insect cells: Huprine-based affinity purification and crystal structure. FEBS J..

[36] Kabsch W. (2010). XDS. Acta Crystallogr. D Biol. Crystallogr..

[37] McCoy A.J., Grosse-Kunstleve R.W., Adams P.D., Winn M.D., Storoni L.C., Read R.J. (2007). Phaser crystallographic software. J. Appl. Crystallog..

[38] Emsley P., Lohkamp B., Scott W.G., Cowtan K. (2010). Features and development of Coot. Acta Crystallogr. D Biol. Crystallogr..

[39] Adams P.D., Afonine P.V., Bunkóczi G., Chen V.B., Davis I.W., Echols N., Headd J.J., Hung L.W., Kapral G.J., Grosse-Kunstleve R.W. (2010). PHENIX: A comprehensive Python-based system for macromolecular structure solution. Acta Crystallogr. D Biol. Crystallogr..

[40] Hagenow S., Stasiak A., Ramsay R.R., Stark H. (2017). Ciproxifan, a histamine H_3_ receptor antagonist, reversibly inhibits monoamine oxidase A and B. Sci. Rep..

[41] Ou B., Hampsch-Woodill M., Prior R.L. (2001). Development and validation of an improved oxygen radical absorbance capacity assay using fluorescein as the fluorescent probe. J. Agric. Food Chem..

[42] Mura F., Silva T., Castro C., Borges F., Zuniga M.C., Morales J., Olea-Azar C. (2014). New insights into the antioxidant activity of hydroxycinnamic and hydroxybenzoic systems: Spectroscopic, electrochemistry, and cellular studies. Free Radic. Res..

[43] Garrido J., Gaspar A., Garrido E.M., Miri R., Tavakkoli M., Pourali S., Saso L., Borges F., Firuzi O. (2012). Alkyl esters of hydroxycinnamic acids with improved antioxidant activity and lipophilicity protect PC12 cells against oxidative stress. Biochimie.

[44] Fernandes C., Pinto M., Martins C., Gomes M.J., Sarmento B., Oliveira P.J., Remião F., Borges F. (2018). Development of a PEGylated-Based Platform for Efficient Delivery of Dietary Antioxidants Across the Blood-Brain Barrier. Bioconjug. Chem..

[45] Camurri G., Zaramella A. (2001). High-throughput liquid chromatography/mass spectrometry method for the determination of the chromatographic hydrophobicity index. Anal. Chem..

[46] Valko K., Nunhuck S., Bevan C., Abraham M.H., Reynolds D.P. (2003). Fast gradient HPLC method to determine compounds binding to human serum albumin. Relationships with octanol/water and immobilized artificial membrane lipophilicity. J. Pharm. Sci..

[47] Anand P., Singh B. (2013). A review on cholinesterase inhibitors for Alzheimer’s disease. Arch. Pharm. Res..

[48] Seltzer B. (2005). Donepezil: A review. Expert Opin. Drug Metab. Toxicol..

[49] Kumar A., Pintus F., Di Petrillo A., Medda R., Caria P., Matos M.J., Vina D., Pieroni E., Delogu F., Era B. (2018). Novel 2-pheynlbenzofuran derivatives as selective butyrylcholinesterase inhibitors for Alzheimer’s disease. Sci. Rep..

[50] Karlsson D., Fallarero A., Brunhofer G., Mayer C., Prakash O., Mohan C.G., Vuorela P., Erker T. (2012). The exploration of thienothiazines as selective butyrylcholinesterase inhibitors. Eur. J. Pharm. Sci..

[51] Moorad D.R., Luo C., Saxena A., Doctor B.P., Garcia G.E. (1999). Purification and determination of the amino acid sequence of equine serum butyrylcholinesterase. Toxicol. Methods.

[52] Afonine P.V., Moriarty N.W., Mustyakimov M., Sobolev O.V., Terwilliger T.C., Turk D., Urzhumtsev A., Adams P.D. (2015). FEM: Feature-enhanced map. Acta Crystallogr. D Biol. Crystallogr..

[53] Nicolet Y., Lockridge O., Masson P., Fontecilla-Camps J.C., Nachon F. (2003). Crystal structure of human butyrylcholinesterase and of its complexes with substrate and products. J. Biol. Chem..

[54] Li H., Deng Z., Wu T., Liu R., Loewen S., Tsao R. (2012). Microwave-assisted extraction of phenolics with maximal antioxidant activities in tomatoes. Food Chem..

[55] Carp O.E., Moraru A., Pinteala M., Arvinte A. (2021). Electrochemical behaviour of piperine. Comparison with control antioxidants. Food Chem..

[56] Silva F.S., Starostina I.G., Ivanova V.V., Rizvanov A.A., Oliveira P.J., Pereira S.P. (2016). Determination of Metabolic Viability and Cell Mass Using a Tandem Resazurin/Sulforhodamine B Assay. Curr. Protoc. Toxicol..

[57] Repetto G., del Peso A., Zurita J.L. (2008). Neutral red uptake assay for the estimation of cell viability/cytotoxicity. Nat. Protoc..

[58] Chavarria D., Fernandes C., Silva V., Silva C., Gil-Martins E., Soares P., Silva T., Silva R., Remiao F., Oliveira P.J. (2020). Design of novel monoamine oxidase-B inhibitors based on piperine scaffold: Structure-activity-toxicity, drug-likeness and efflux transport studies. Eur. J. Med. Chem..

[59] Valko K., Bevan C., Reynolds D. (1997). Chromatographic Hydrophobicity Index by Fast-Gradient RP-HPLC: A High-Throughput Alternative to log P/log D. Anal. Chem..

[60] Hitchcock S.A., Pennington L.D. (2006). Structure-brain exposure relationships. J. Med. Chem..

[61] Pajouhesh H., Lenz G.R. (2005). Medicinal Chemical Properties of Successful Central Nervous System Drugs. NeuroRx.

[62] Valko K., Du C.M., Bevan C., Reynolds D.P., Abraham M.H. (2001). Rapid method for the estimation of octanol/water partition coefficient (Log Poct) from gradient RP-HPLC retention and a hydrogen bond acidity term (∑α2H). Curr. Med. Chem..

[63] Smith R.A.J., Porteous C.M., Gane A.M., Murphy M.P. (2003). Delivery of bioactive molecules to mitochondria in vivo. Proc. Natl. Acad. Sci. USA.

