# Supplementary material for: Fine-Tuning the Biological Profile of Multitarget Mitochondriotropic Antioxidants for Neurodegenerative Diseases
Source: Antioxidants (Basel). 2021 Feb 23;10(2):329. doi: 10.3390/antiox10020329 (PMC7926627; doi:10.3390/antiox10020329)
Supplement: Supplementary file 1 [file antioxidants-10-00329-s001.pdf]

Electronic Supplementary Information

Fine-tuning the biological profile of multitarget mitochondriotropic antioxidants for neurodegenerative diseases

Daniel Chavarria‡[1], Ophélie Da Silva[2], Sofia Benfeito‡[1], Sandra Barreiro[3], Jorge Garrido[1,4], Fernando Cagide[1], Pedro Soares[1], Fernando Remião[3], Xavier Brazzolotto[2], Florian Nachon[2], Paulo Jorge Oliveira[5], José Dias[2], Fernanda Borges[1*]

[1] CIQUP/Department of Chemistry and Biochemistry, Faculty of Sciences, University of Porto, 4169-007 Porto, Portugal

[2] Département de Toxicologie et Risques Chimiques, Institut de Recherche Biomédicale des Armées, 91223, Brétigny-sur-Orge, France

[3] UCIBIO-REQUIMTE, Laboratory of Toxicology, Department of Biological Sciences, Faculty of Pharmacy, University of Porto, Porto, Portugal

[4] CIQUP/Department of Chemical Engineering, School of Engineering (ISEP), Polytechnic of Porto, 4200-072 Porto, Portugal.

[5] CNC - Center for Neuroscience and Cell Biology, University of Coimbra, UC Biotech, Biocant Park, Cantanhede, Portugal.

‡ These authors contributed equally.

* Corresponding Author:

Fernanda Borges, CIQUP/Department of Chemistry and Biochemistry, Faculty of Sciences, University of Porto, Porto 4169-007, Portugal. E-mail: fborges@fc.up.pt

Contents of Electronic Supporting information:

1. Experimental section 3
1.1. Chemistry 3
1.1.1. Reagents 3
1.1.2. Materials and apparatus 3
1.2. Enzymatic assays 3
1.2.1. Materials and general conditions 3
1.2.2. Evaluation of human monoamine oxidase (*h*MAO) inhibitory activity 4
1.2.3. Evaluation of eel acetylcholinesterase and equine butyrylcholinesterase inhibitory activity 4
1.3. Oxygen radical absorbance capacity (ORAC-FL) assay 5
1.4. Electrochemical measurements 6
1.5. *In vitro* toxicology 6
1.5.1. Materials 6
1.5.2. Cell culture 6
1.5.3. Cytotoxicity assays 7
1.5.3.1. Resazurin reduction assay 7
1.5.3.2. Neutral red uptake assay 7
1.6. Evaluation of the chromatographic hydrophobicity index 8
2. Tables 9
3. Figures 10
4. References 15

1. Experimental section

1.1. Chemistry

1.1.1. Reagents

Piperine, sodium hydroxide (NaOH), *N*-(4-bromobutyl)phthalimide, *N*-(6-bromohexyl)phthalimide, triphenylphosphine (TPP), *n*-butylamine, piperine, sodium hydroxide (NaOH), triethylamine (Et_3N), ethyl chloroformate, and boron tribromide dimethyl sulfide complex ($BBr_3.S(CH_3)_2$) were purchased from Sigma Aldrich (St. Louis, MO, USA), Alfa-Aesar (Kandel, Germany) or Fluorochem (Hadfield, UK). All other reagents and solvents were pro analysis grade and were acquired from Carlo Erba Reagents (Val de Reuil, France) and Scharlab and were used without additional purification.

1.1.2. Materials and apparatus

The monitoring of reactions progress was performed by thin layer chromatography (TLC) on precoated silica gel 60 F254 acquired from Merck (Darmstad, Germany). TLC spots were visualized under UV detection (254 nm and 365 nm). Flash column chromatography was carried out on silica gel 60 Å acquired from Carlo-Erba Reactifs (SDS, France). The fractions with the desired compound were gathered and concentrated *in vacuo*. The solvents were evaporated using a Buchi Rotavapor.

^{1}H and ^{13}C NMR and DEPT135 data were acquired, at room temperature, on a Brüker AMX 300 spectrometer operating at 400 and 100 MHz, respectively. The solvents used in NMR experiments included $CDCl_3$-d_1, MeOD-d_4 and DMSO-d_6. The chemical shifts are expressed in δ (ppm) values relative to tetramethylsilane (TMS) as an internal reference and coupling constants (*J*) are given in Hz. Carbon signals present in DEPT135 spectra were underlined. Electrospray ionization mass spectra (ESI-MS) were carried out on a Bruker Microtof apparatus or on a LTQ Orbitrap™ XL hybrid mass spectrometer (Thermo Fischer Scientific, Bremen, Germany) controlled by *LTQ Tune Plus 2.5.5* and *Xcalibur 2.1.0*. The data are reported as m/z (% of the relative intensity of the most important fragments).

1.2. Enzymatic assays

1.2.1. Materials and general conditions

Microsomal MAO isoforms prepared from insect cells (BTI-TN-5B1-4) infected with recombinant baculovirus containing cDNA inserts for *h*MAO-A or *h*MAO-B, kynuramine, (*R*)-(−)-deprenyl, rasagiline, safinamide and clorgyline were purchased from Sigma Aldrich.

Eel acetylcholinesterase (*ee*AChE, E.C. 3.1.1.7, from *Electrophorus electricus*), equine butyrylcholinesterase (*eq*BuChE, E.C. 3.1.1.8, from equine serum), DTNB, acetylthiocholine iodide (ATCI), and butylthiocholine iodide (BTCI) were purchased from Sigma Aldrich. Enzyme and DTNB solutions were prepared in sodium phosphate-buffered conditions (50 mM, pH = 7.4).

Substrate solutions (ATCI or BCTI) were prepared in deionized water (conductivity < 0.1 μS.cm^{-1}).

1.2.2. Evaluation of human monoamine oxidase (*h*MAO) inhibitory activity

The human monoamine oxidase (*h*MAO) inhibitory outline was assessed in microsomal MAO isoforms prepared from insect cells (BTI-TN-5B1-4) infected with recombinant baculovirus containing cDNA inserts for *h*MAO-A or *h*MAO-B and by measuring the enzymatic conversion rates of kynuramine into 4-hydroxyquinoline. The appropriate amounts of *h*MAO-A and *h*MAO-B were adjusted to obtain, in our experimental conditions, the same maximum velocity (V_{max} = 50 pmol.min^{-1}) for both isoforms (*h*MAO-A: 3 ng.μL^{-1}; *h*MAO-B: 12 ng.μL^{-1}). All assays were performed in sodium phosphate buffer solution 50 mM pH 7.4.

The compounds under study and reference inhibitors were pre-incubated at 37 ºC for 10 min in the presence of kynuramine (K_m *h*MAO-A = 20 μM; K_m *h*MAO-B = 20 μM; final concentration: 2×K_m) in 96-well microplates (BRANDplates, pureGrade^{TM}, BRAND GMBH, Wertheim, Germany). Then, the reaction was started with the addition of *h*MAO-A or *h*MAO-B. Initial velocities were determined spectrophotometrically in a microplate reader (BioTek Synergy HT from BioTek Instruments, Winooski, VT, USA) at 37 °C by measuring the formation of 4-hydroxyquinoline at 316 nm, over at least 30 min (interval of 1 min). Data were analysed using GraphPad PRISM version 6 for Windows (GraphPad Software®, San Diego, CA, USA). The initial velocities, obtained from the linear phase of product formation, were normalized and plotted against the respective inhibitor concentration. IC_{50} values were obtained from dose-response curves and were expressed as mean ± standard deviation. IC_{50} values were determined from at least three independent experiments, each performed in triplicate.

1.2.3. Evaluation of eel acetylcholinesterase and equine butyrylcholinesterase inhibitory activity

The *ee*AChE and *eq*BChE inhibitory activities of the compounds under study were evaluated following the Ellman's method [1, 2]. Briefly, a mixture containing sodium phosphate buffer, enzyme (final concentration: *ee*AChE 0.05 U/mL and *ee*BChE 0.05 U/mL), DTNB (final concentration: 400 μM) and the test compounds/reference inhibitors were preincubated for 5 minutes at 30 ºC in a 96-well microplate (BRANDplates, pureGradeTM, BRAND GMBH, Wertheim, Germany). In control experiments, sodium phosphate buffer was used instead of the compound solution. Then, the substrate solution (final concentration: ATCI 150 μM; BTCI 400 μM) was added, and the absorbance values at 412 nm were recorded minutely for 5 minutes at 30 ºC in a microplate reader (BioTek Synergy HT from BioTek Instruments, Winooski, VT, USA). The rates of the substrate enzymatic hydrolysis were determined, normalized and plotted against the respective inhibitor concentration. IC_{50} values were obtained from the dose-response curves

and were expressed as mean ± standard deviation. IC_{50} values were determined in at least three independent experiments, each performed at least in triplicates.

1.3. Oxygen radical absorbance capacity (ORAC-FL) assay

The ORAC-FL analyses were performed on a multiplate reader (BioTek Synergy HT from BioTek Instruments, Winooski, VT, USA) using black polystyrene 96-well plates. Fluorescence was measured from the top, at an excitation wavelength of 485/20 nm and an emission at 528/20 nm. The plate reader was controlled by Gen5 software. 2,2'-Azobis-(amidinopropane) dihydrochloride (AAPH), (±)-6-hydroxy-2,5,7,8-tetramethylchromane-2-carboxylic acid (trolox) and fluorescein (FL) were purchased from Sigma Aldrich. The general procedure was adapted from the literature [3, 4], with some modifications.

The reaction was carried out in 75 mM of sodium phosphate buffer solution (pH = 7.4) at a final volume of 200 µL. Stock solutions of FL (5 mM) and antioxidants (5 mM) were prepared in ethanol due to their low solubility in buffer at high concentrations. Then, they were diluted in sodium phosphate buffer solution (pH = 7.4). The range of antioxidant concentrations was empirically selected to obtain a good separation between the fluorescence curves. The test compounds (final concentrations range of 0.1-2 µM) and fluorescein (40 nM, final concentration) were placed in each well and pre-incubated for 15 min at 37 ºC before the addition of AAPH (final concentration 18 mM). The microplate was immediately placed in the reader and automatically shaken prior to each reading. The fluorescence was measured minutely for 120 min. A control assay with fluorescein, AAPH and buffer (instead of the antioxidant solution) was performed for each assay. Trolox was also included in each assay as reference antioxidant in a final concentration range of 2-8 µM. All reaction mixtures were prepared in triplicate and at least three independent experiments were performed for each test compound. The fluorescence decay curves were normalized by the maximum value of fluorescence, and the areas under the curves (AUC) were determined according to the following **Equation 2**.

$$\text{AUC} = 1 + \sum_{i=1}^{i=120} \frac{f_i}{f_0} = 1 + \frac{f_1}{f_0} + \frac{f_2}{f_0} + \frac{f_3}{f_0} + \ldots + \frac{f_{120}}{f_0} \quad \textbf{(Equation 2)}$$

where f_0 = initial fluorescence reading at 0 min and f_i = fluorescence reading at time i. The net AUC is obtained by subtracting the AUC of the blank from that of a sample. The inhibition capacity, expressed as the relative Trolox equivalent ORAC values, was quantified using **Equation 3**.

$$\text{relative ORAC value} = \frac{AUC_{antioxidant} - AUC_{control}}{AUC_{trolox} - AUC_{control}} \times \frac{[\text{Trolox}]}{[\text{antioxidant}]} \quad \textbf{(Equation 3)}$$

where $AUC_{control}$ = area under the curve of control; $AUC_{antioxidant}$ = area under the curve of the test compound; AUC_{trolox} = area under the curve of Trolox (reference); [trolox] = Trolox concentration; and [antioxidant] = antioxidant concentration.

1.4. Electrochemical measurements

Voltammetric studies were carried out using an Autolab PGSTAT 12 potentiostat/galvanostat (Metrohm-Autolab, Netherlands) and one compartment glass electrochemical cell. The voltammetric data were acquired at room temperature using a three-electrode system: a glassy carbon working electrode (GCE) (d = 2 mm), a platinum wire counter electrode and an Ag/AgCl saturated KCl reference electrode (Metrohm, Switzerland). The working electrode was polished manually with an aqueous slurry of alumina powder (BDH Chemicals, VWR, USA) on a microcloth pad and rinsed with water before use. A Crison pH-meter with glass electrode was used for the pH measurements (Crison, Barcelona, Spain).

Differential pulse voltammetry (DPV) experiments were performed as described elsewhere [5]. Test solutions were prepared directly in the electrochemical cell, by diluting ethanolic solutions of the compounds under study (10 mM, 100 μL) in the supporting electrolyte (10 mL) to obtain a final concentration of 100 μM. The scan rate used in differential pulse voltammetry (DPV) was 5 $mV.s^{-1}$.

1.5. *In vitro* toxicology

1.5.1. Materials

All reagents used were of analytical grade or of the highest grade available. Neutral red (NR) solution, resazurin, Dulbecco's Modified Eagle's Medium (DMEM) with 4.5 g/L glucose, retinoic acid, 12-*O*-tetradecanoylphorbol-13-acetate (TPA), 2',7'-dichorofluorescein diacetate (DCFH-DA), Rhodamine 123 (RHO 123) and zosuquidar (ZOS) were obtained from Sigma Aldrich. Reagents used in cell culture such as heat-inactivated fetal bovine serum (FBS), antibiotic (10,000 U/mL penicillin, 10,000 μg/mL streptomycin), MEM Non-Essential Amino Acids solution (100×) (MEM NEAA), Hanks' balanced salt solution (HBSS) with or without calcium and magnesium (HBSS(+/+) or HBSS (-/-), respectively) and 0.25% trypsin/1 mM ethylenediamine tetraacetic acid (EDTA) were purchased from Gibco Laboratories (Lenexa, KS, USA). Dimethylsulfoxide (DMSO), absolute ethanol, sodium bicarbonate and acetic acid were obtained from Merck (Darmstadt, Germany). Triton™ X-100 detergent solution was acquired from Thermo Fisher Scientific (Waltham, MA, USA).

1.5.2. Cell culture

Human neuroblastoma SH-SY5Y cells were routinely cultured into 75 cm^3 flasks and maintained in DMEM with 4.5 g/L glucose, supplemented with 10 % heat-inactivated FBS (v/v), 1 % MEM NEAA (v/v) and 1% penicillin/streptomycin (v/v). Cells were maintained at 37 °C in a humidified, 5% CO_2–95% air atmosphere, and the cell culture medium was changed every 2-3

days. The cultures were passaged once a week by trypsinization (0.25 % trypsin/1 mM EDTA). To avoid phenotypic changes, the cells used for all experiments were taken between 19th and 28th passages. Cells were differentiated as described previously by Fernandes *et al*. [6]. Briefly, SH-SY5Y cells were seeded onto 96-well plates at a density of 25,000 cells/cm^2 in cell culture medium with *trans*-retinoic acid (RA) (final concentration of 10 μM) and incubated for 3 days at 37 °C in a humidified, 5% CO_2–95% air atmosphere. The cell culture medium was then removed and replaced with medium supplemented with 12-*O*-tetradecanoylphorbol-13-acetate (TPA) (final concentration 80 nM), and cells were incubated for 3 days. Stock solutions of RA (10 mM) and TPA (80 μM) were prepared in DMSO.

1.5.3. Cytotoxicity assays

1.5.3.1. Resazurin reduction assay

Resazurin is a non-fluorescent, non-toxic water-soluble dye able to permeate through cell membranes [7]. Resazurin is enzymatically reduced inside the cells into the pink-colored and highly fluorescent resorufin [8, 9]. Since the conversion of resazurin into resorufin occurs mostly in mitochondria, the amount of resorufin produced can be used as indicator of metabolic activity [10]. The quantity of resorufin production is proportional to the number of viable cells in culture and can be quantified fluorometrically or colorimetrically [8].

Resazurin reduction assay was performed as previously described by Fernandes *et al.* [6]. Briefly, after the 24 h incubation periods with the test compounds, the cell culture medium was removed and replaced with fresh culture medium with resazurin (10 μg/mL) prepared in cell culture medium. Differentiated SH-SY5Y cells were incubated for 2 h at 37 °C in a humidified 5% CO_2–95% air atmosphere. Then, the fluorescence was read in a multiwell microplate reader (PowerWave XS from BioTek Instruments, Vermont, US), using an excitation wavelength of 540 nm and an emission wavelength of 590 nm. The results were expressed as the percentage of resazurin reduction relative to that of the control (non-treated) cells [resazurin reduction (% of control)] ± standard error mean (SEM) of at least three independent experiments.

1.5.3.2. Neutral red uptake assay

The neutral red (NR) uptake assay is based on the ability of viable cells to incorporate the supravital dye NR and retain it inside the lysosomes [11]. NR uptake assay was performed as previously described by Fernandes *et al*. [12] Briefly, after the 24 h incubation periods with the test compounds, the cell culture medium was removed and NR solution (50 mg/mL) in fresh cell culture medium was added. Differentiated SH-SY5Y cells and Caco-2 cells were incubated for 2 h and 1 h, respectively, at 37 °C in a humidified, 5% CO_2–95% air atmosphere. Then, the cell culture medium was removed and replaced by ethanol absolute/distilled water (1:1) with 5 % acetic acid to liberate the dye from the viable cells. The absorbance was measured at 540 nm in a

multiwell microplate reader (PowerWave XS from BioTek Instruments). The results were expressed as the percentage of NR uptake relative to that of the control (non-treated) cells [NR uptake (% of control)] ± standard error mean (SEM) of at least three independent experiments.

1.6. Evaluation of the chromatographic hydrophobicity index

Chromatographic hydrophobicity indexes (CHIs) values were determined using the retention times (t_R) of samples and a mixture of reference compounds. The data was acquired on a Shimadzu high-performance liquid chromatograph SPD-M20A system (Shimadzu, Kyoto, Japan) with a Luna C18 (2) column (Phenomenex, CA, USA) 150×4.6 mm, 5 μm. In CHI determinations at pH 2.3, mobile phase A was aqueous solution of acetic acid 1 % (v/v) (pH 2.3), and mobile phase B was acetonitrile. The following gradient program was applied: 1 mL/min flow, rt, injection volume 20 μL, gradient 0−6 min 0-100 % B, 6−14 min 100% B, 14−16 min 100-0 % B.

Calibration plots were obtained using a mixture of the following compounds: paracetamol, theophylline, caffeine, benzimidazole, colchicine, carbamazepine, indole, propiophenone, butyrophenone, valerophenone, and heptanophenone (**Table S1**, **Figure S2**). Stock solutions of the test compounds (10 μM) were prepared in DMSO and diluted in acetonitrile/water (1:1) to obtain a final concentration of 250 μM. Chromatographic hydrophobicity index (CHI) values were calculated as previously described [13, 14].

2. Tables

Table S1. Retention times (t_R) of the standard mixture obtained by LC/UV at pH 2.3. CHI_0 values at pH 2.3 were taken from Camurri *et al.* [13].

Compounds	t_R(min)	CHI_0 pH 2.3
**Benzimidazole**	6.769	6.30
**Theophyline**	7.357	17.9
**Paracetamol**	7.499	18.77
**Caffeine**	7.643	23.41
**Colchicine**	8.496	43.9
**Carbamazepine**	9.370	60.42
**Indole**	10.266	72.1
**Propiophenone**	10.618	77.4
**Butyrophenone**	11.277	87.3
**Valerophenone**	11.967	96.4
**Heptanophenone**	13.535	112.1

3. Figures

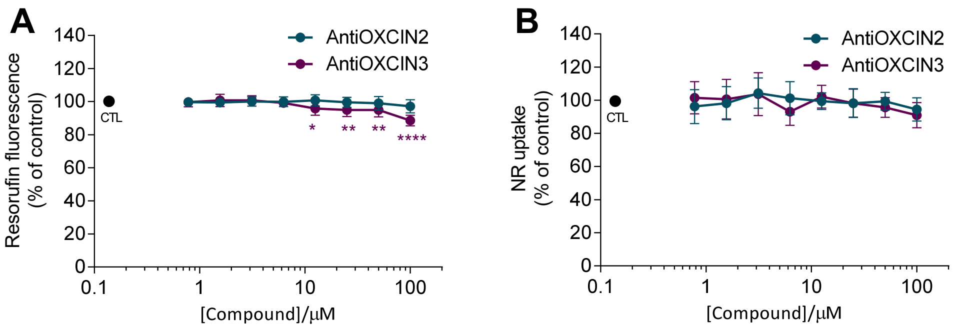

Figure S1. Cellular viability of differentiated SH-SY5Y neuroblastoma cells after a 24 h treatment with **AntiOXCIN2** and **AntiOXCIN3** at eight different concentrations (0.80-100 μM). Cellular viability was evaluated using the resazurin reduction assay (**A**) and the NR uptake assay (**B**). Results are expressed as the mean % of untreated controls ± SEM (n = 3). Statistical comparisons were made using two-way ANOVA followed by Dunnet's multiple comparison test. In all cases, p values lower than 0.05 were considered significant (* $p < 0.05$, ** $p < 0.01$, *** $p < 0.001$, **** $p < 0.0001$ vs untreated cells).

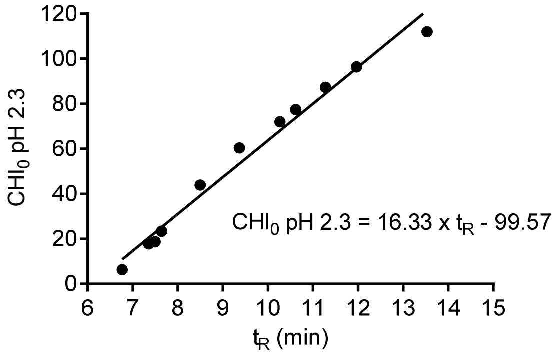

Figure S2. Linear correlation obtained by plotting the retention times (t_R) of each of the individual standard mixture compounds against the CHI values at pH 2.3 (CHI_0 pH 2.3) reported by Camurri et al. [13].

Compound 2

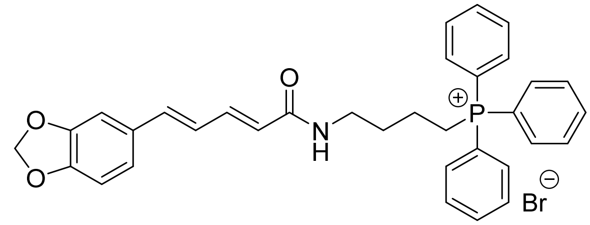

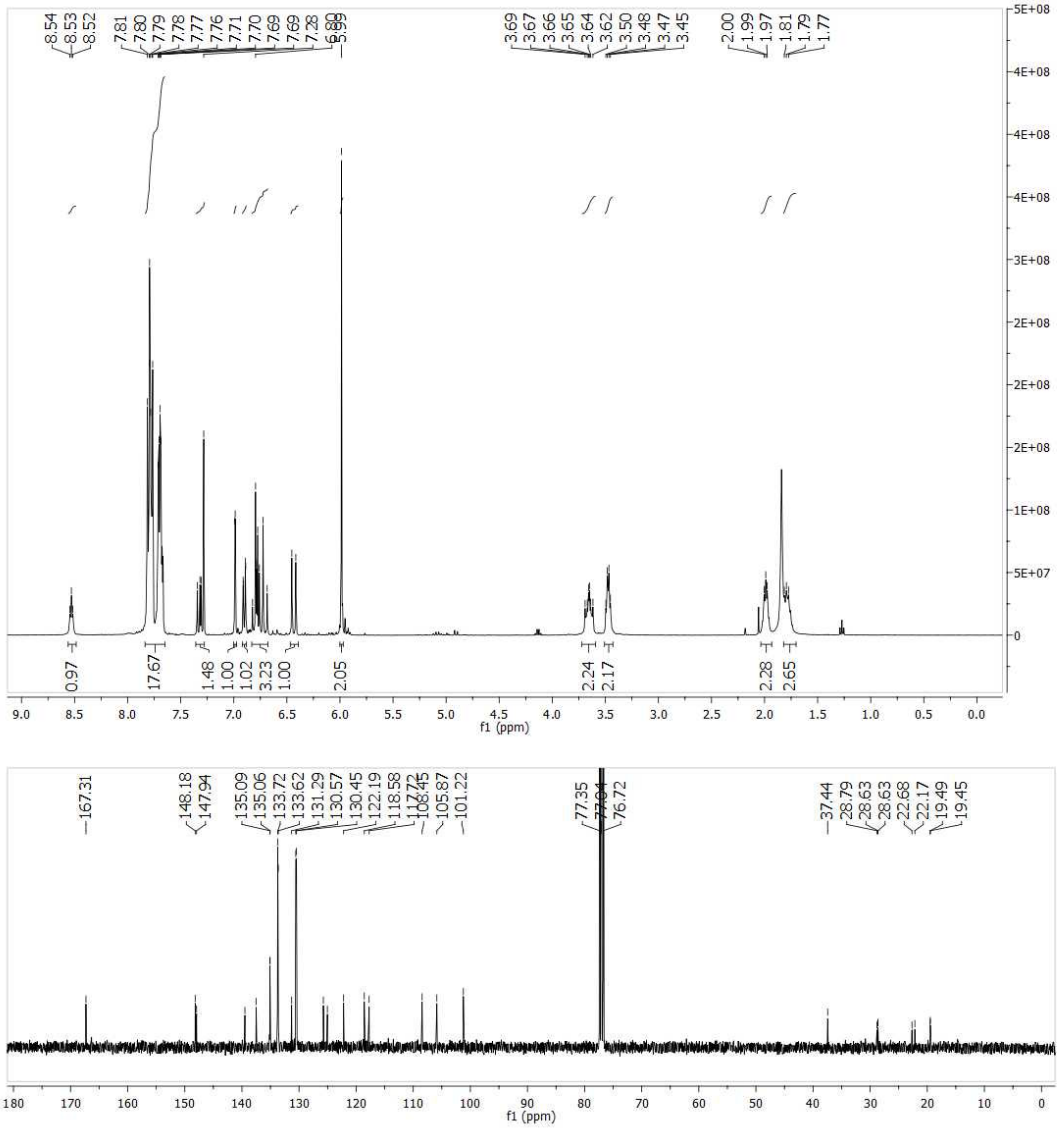

Figure S3. ^{1}H and ^{13}C NMR spectra of compound **2** (NMR spectra obtained in $CDCl_3$-d_1).

Compound 3

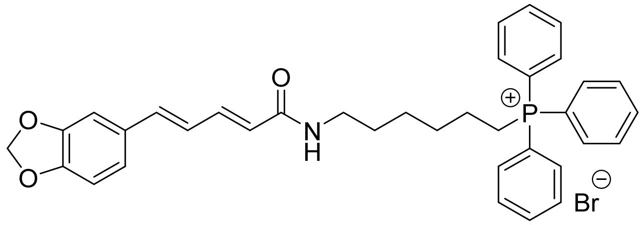

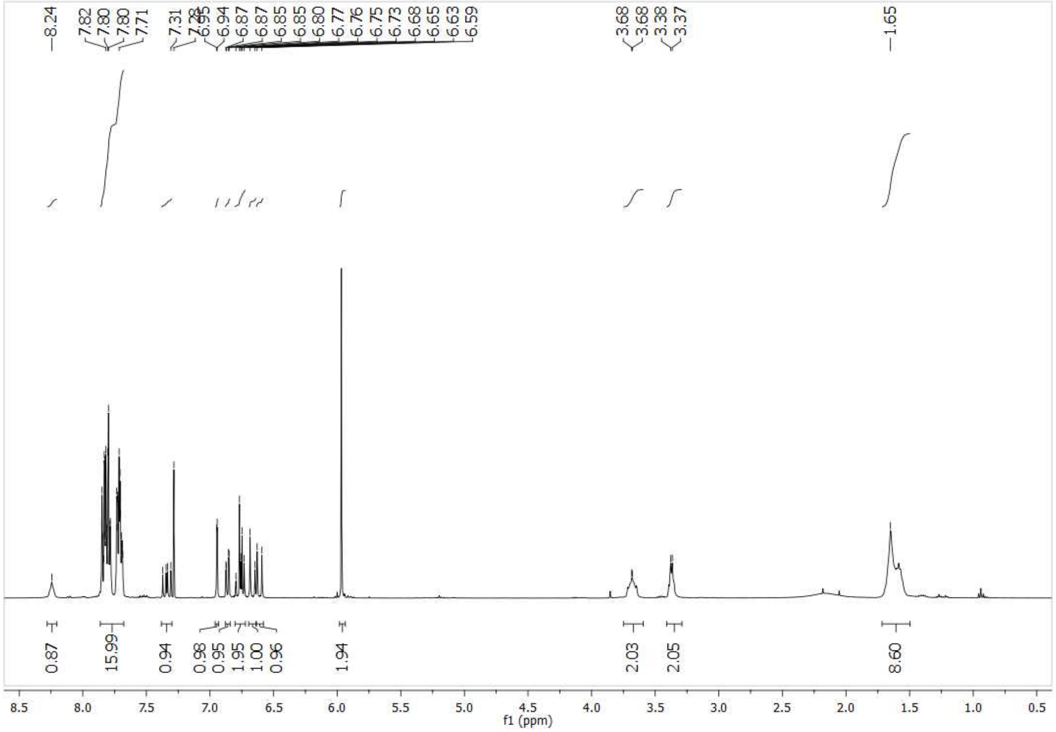

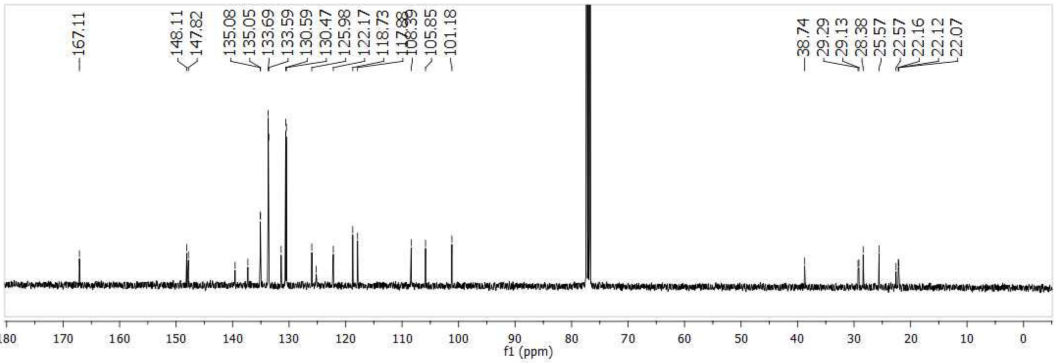

Figure S4. ^{1}H and ^{13}C NMR spectra of compound **3** (NMR spectra obtained in $CDCl_3$-d_1).

Compound 4

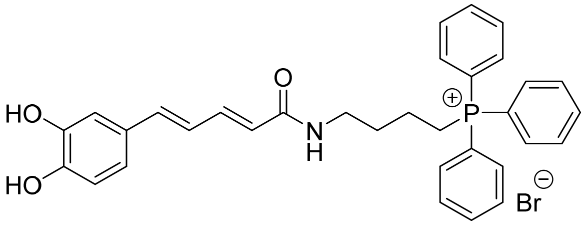

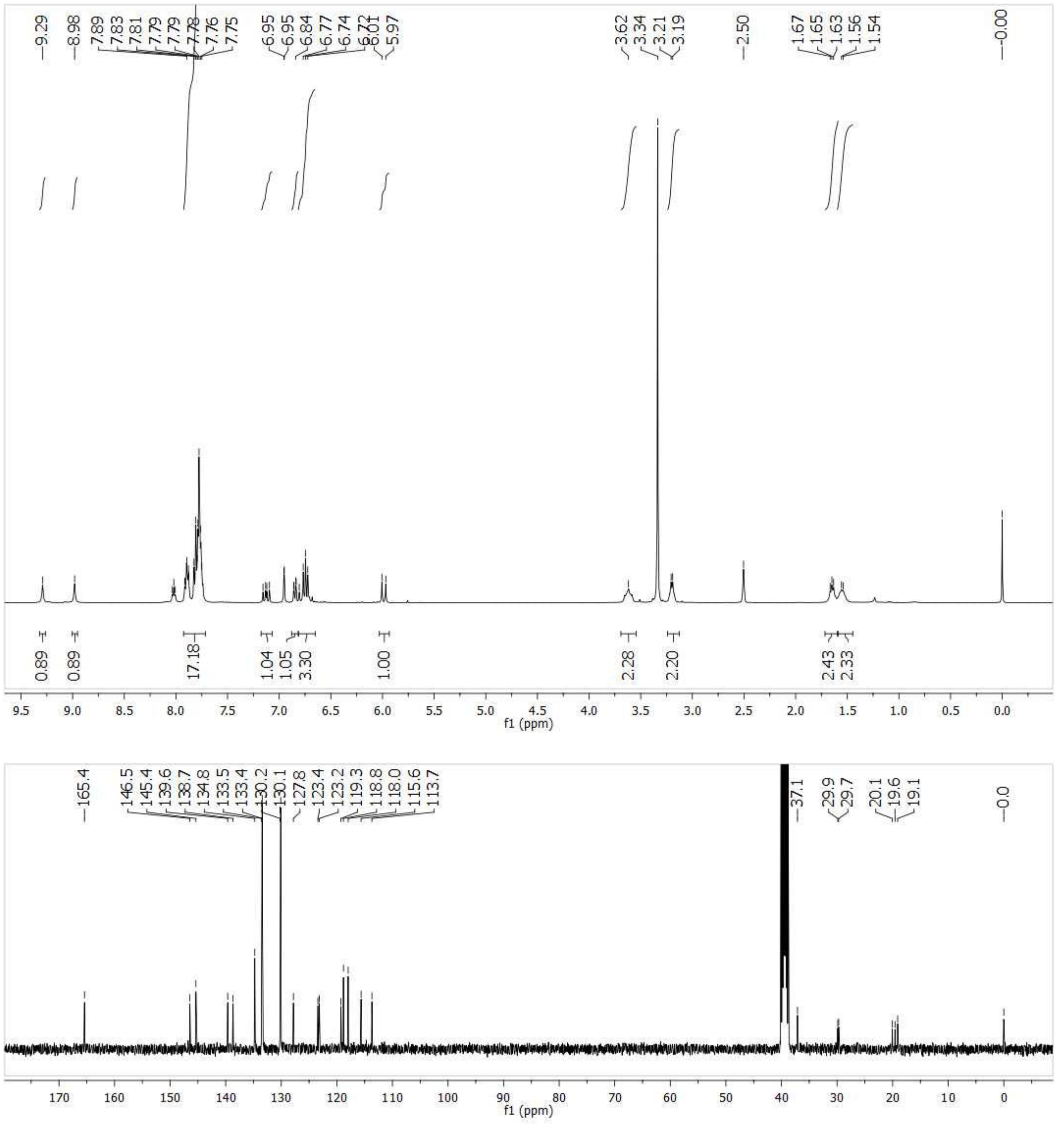

Figure S5. ^{1}H and ^{13}C NMR spectra of compound 4 (NMR spectra obtained in DMSO-d_6).

Compound 5

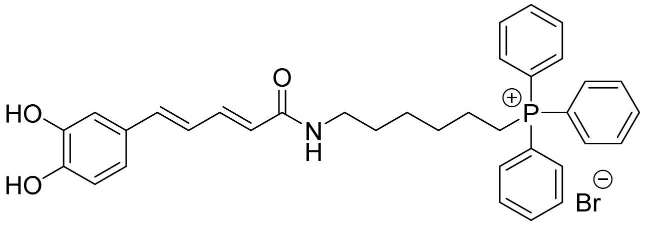

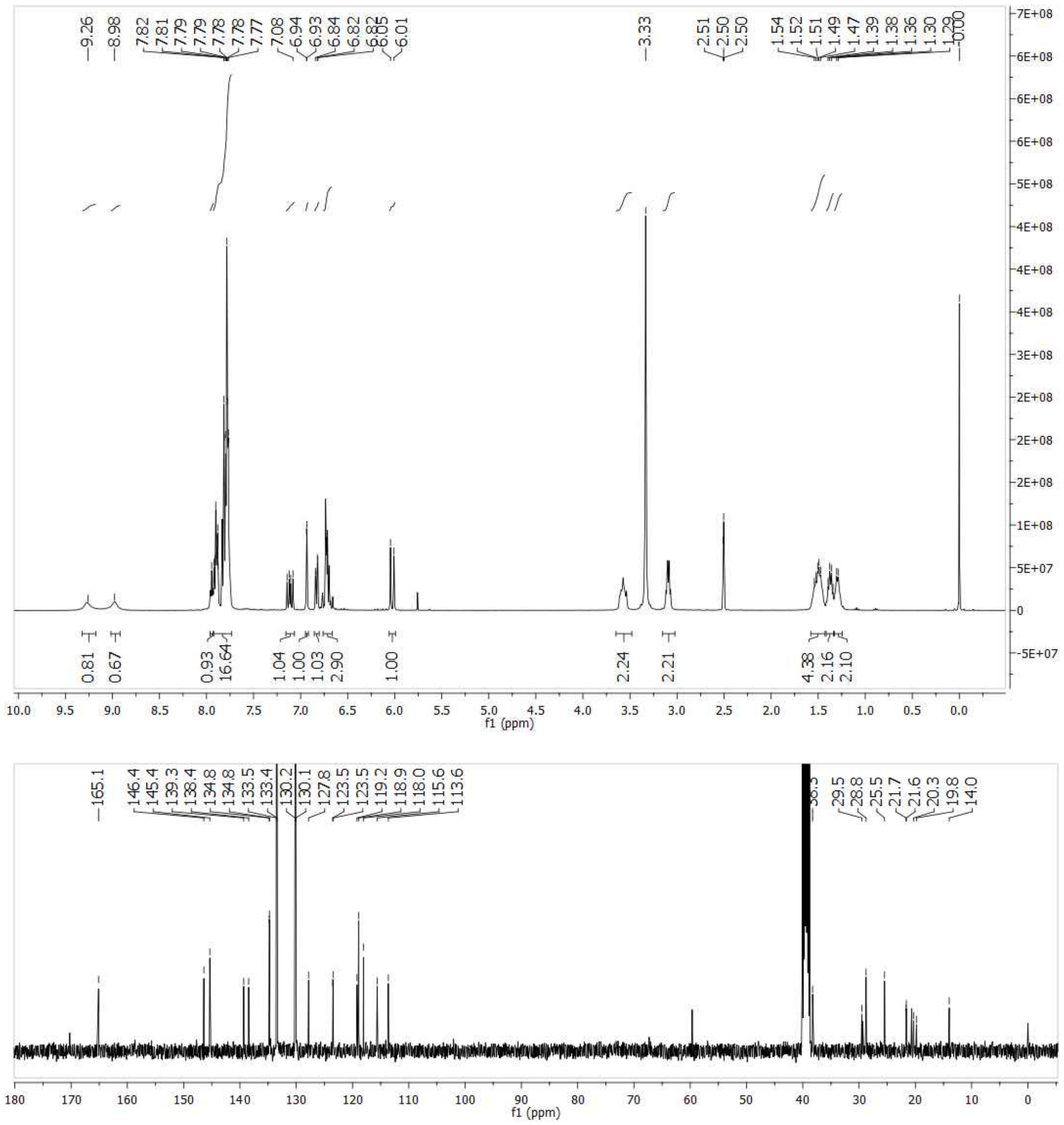

Figure S6. ^{1}H and ^{13}C NMR spectra of compound **5** (NMR spectra obtained in DMSO-d_6).

4. References

[1] G.L. Ellman, K.D. Courtney, V. Andres, Jr., R.M. Feather-Stone, A new and rapid colorimetric determination of acetylcholinesterase activity, Biochem. Pharmacol., 7 (1961) 88-95. https://doi.org/10.1016/0006-2952(61)90145-9

[2] S. Di Giovanni, A. Borloz, A. Urbain, A. Marston, K. Hostettmann, P.A. Carrupt, M. Reist, *In vitro* screening assays to identify natural or synthetic acetylcholinesterase inhibitors: thin layer chromatography versus microplate methods, Eur. J. Pharm. Sci., 33 (2008) 109-119. https://doi.org/10.1016/j.ejps.2007.10.004

[3] B. Ou, M. Hampsch-Woodill, R.L. Prior, Development and validation of an improved oxygen radical absorbance capacity assay using fluorescein as the fluorescent probe, J. Agric. Food Chem., 49 (2001) 4619-4626. https://doi.org/10.1021/jf010586o

[4] F. Mura, T. Silva, C. Castro, F. Borges, M.C. Zuniga, J. Morales, C. Olea-Azar, New insights into the antioxidant activity of hydroxycinnamic and hydroxybenzoic systems: spectroscopic, electrochemistry, and cellular studies, Free Radic. Res., 48 (2014) 1473-1484. https://doi.org/10.3109/10715762.2014.965702

[5] J. Garrido, A. Gaspar, E.M. Garrido, R. Miri, M. Tavakkoli, S. Pourali, L. Saso, F. Borges, O. Firuzi, Alkyl esters of hydroxycinnamic acids with improved antioxidant activity and lipophilicity protect PC12 cells against oxidative stress, Biochimie, 94 (2012) 961-967. https://doi.org/10.1016/j.biochi.2011.12.015

[6] C. Fernandes, M. Pinto, C. Martins, M.J. Gomes, B. Sarmento, P.J. Oliveira, F. Remião, F. Borges, Development of a PEGylated-Based Platform for Efficient Delivery of Dietary Antioxidants Across the Blood-Brain Barrier, Bioconjug. Chem., 29 (2018) 1677-1689. https://doi.org/10.1021/acs.bioconjchem.8b00151

[7] S.N. Rampersad, Multiple applications of Alamar Blue as an indicator of metabolic function and cellular health in cell viability bioassays, Sensors (Basel), 12 (2012) 12347-12360. https://doi.org/10.3390/s120912347

[8] J. O'Brien, I. Wilson, T. Orton, F. Pognan, Investigation of the Alamar Blue (resazurin) fluorescent dye for the assessment of mammalian cell cytotoxicity, Eur. J. Biochem., 267 (2000) 5421-5426. https://doi.org/10.1046/j.1432-1327.2000.01606.x

[9] E.M. Czekanska, Assessment of cell proliferation with resazurin-based fluorescent dye, Methods Mol. Biol., 740 (2011) 27-32. https://doi.org/10.1007/978-1-61779-108-6_5

[10] A. van Tonder, A.M. Joubert, A.D. Cromarty, Limitations of the 3-(4,5-dimethylthiazol-2-yl)-2,5-diphenyl-2H-tetrazolium bromide (MTT) assay when compared to three commonly used cell enumeration assays, BMC Res. Notes, 8 (2015) 47. https://doi.org/10.1186/s13104-015-1000-8

[11] G. Repetto, A. del Peso, J.L. Zurita, Neutral red uptake assay for the estimation of cell viability/cytotoxicity, Nat. Protoc., 3 (2008) 1125-1131. https://doi.org/10.1038/nprot.2008.75

[12] C. Fernandes, C. Martins, A. Fonseca, R. Nunes, M.J. Matos, R. Silva, J. Garrido, B. Sarmento, F. Remiao, F.J. Otero-Espinar, E. Uriarte, F. Borges, PEGylated PLGA Nanoparticles As a Smart Carrier to Increase the Cellular Uptake of a Coumarin-Based Monoamine Oxidase B Inhibitor, ACS Appl. Mater. Interfaces, 10 (2018) 39557-39569. https://doi.org/10.1021/acsami.8b17224

[13] G. Camurri, A. Zaramella, High-throughput liquid chromatography/mass spectrometry method for the determination of the chromatographic hydrophobicity index, Anal. Chem., 73 (2001) 3716-3722. https://doi.org/10.1021/ac001388j

[14] K. Valko, S. Nunhuck, C. Bevan, M.H. Abraham, D.P. Reynolds, Fast gradient HPLC method to determine compounds binding to human serum albumin. Relationships with octanol/water and immobilized artificial membrane lipophilicity, J. Pharm. Sci., 92 (2003) 2236-2248. https://doi.org/10.1002/jps.10494
